# Oligomeric states of ASC specks regulate inflammatory responses by inflammasome in the extracellular space

**DOI:** 10.1038/s41420-023-01438-6

**Published:** 2023-04-29

**Authors:** Tae-Geun Yu, Jeong Seok Cha, Gijeong Kim, Yoo-Kyoung Sohn, Youngki Yoo, Uijin Kim, Ji-Joon Song, Hyun-Soo Cho, Hak-Sung Kim

**Affiliations:** 1 Departement of Biological Sciences, Korea Advanced Institute of Science and Technology (KAIST), Daejeon, 34141 Korea; 2 Department of Systems Biology, College of Life Science and Biotechnology, Yonsei University, Seoul, 03722 Korea; 3 Present Address: Research Institute of Pharmacy, Chung-Ang University, Seoul, 06974 Korea; 4 Present Address: R&D Center, Sugentech, Inc., Daejeon, Korea

**Keywords:** Structural biology, Inflammasome, Proteases, Prions, Protein design

## Abstract

Inflammasomes are multi-protein complexes and play a crucial role in host defense against pathogens. Downstream inflammatory responses through inflammasomes are known to be related to the oligomerization degree of ASC specks, but the detailed mechanism still remains unexplored. Here, we demonstrate that oligomerization degrees of ASC specks regulate the caspase-1 activation in the extracellular space. A protein binder specific for a pyrin domain (PYD) of ASC (ASC^PYD^) was developed, and structural analysis revealed that the protein binder effectively inhibits the interaction between PYDs, disassembling ASC specks into low oligomeric states. ASC specks with a low oligomerization degree were shown to enhance the activation of caspase-1 by recruiting and processing more premature caspase-1 through interactions between CARD of caspase-1 (caspase-1^CARD^) and CARD of ASC (ASC^CARD^). These findings can provide insight into controlling the inflammasome-mediated inflammatory process as well as the development of inflammasome-targeting drugs.

## Introduction

Inflammasomes are multi-component protein complexes that play a crucial role in cytosolic host defense against immunological challenges [1, 2]. Inflammasomes are assembled by activation of various pattern-recognition receptors in response to stimuli, triggering the maturation of inflammatory caspase-1 to produce pro-inflammatory cytokines and eventually induce pyroptotic cell death [3]. The regulation of inflammasome activation and downstream signaling is thus critical to appropriate inflammatory responses. However, the dysregulated activation of inflammasomes is linked to various diseases, including Alzheimer’s disease, Parkinson’s disease, peritonitis, sepsis, and type 2 diabetes [4–6].

Assembly of inflammasomes is initiated by activation of sensor proteins, and activated senor proteins recruit and oligomerize the adapter protein apoptosis-associated speck-like protein containing a CARD (ASC) composed of a PYD (pyrin domain) and a CARD (caspase activation and recruitment domain) [7, 8]. The resulting ASC oligomers further form a higher-order structure, called a “speck,” which recruits pro-caspase-1 [9–12]. A proximity-induced auto-proteolysis of premature caspase-1 produces active caspase-1, which is a major effector protein in inflammasomes, leading to the release of cytokines and pyroptotic cell lysis [13, 14]. Following pyroptosis, inflammasome components, including caspase-1 and ASC specks, are released outside the cells, further amplifying inflammatory responses in the extracellular space [15]. The ASC specks have been reported to form in a single foci of cells via an all-or-none process [1, 16]. However, recent studies have revealed that ASC specks with different sizes are generated by various factors [17–19], and the inflammatory responses are affected by the size of ASC specks [9, 18, 20, 21]. In addition, structural changes in ASC specks after cell lysis are inevitable due to the differences between the microenvironment inside and outside the cell. Such observations strongly imply a correlation between the size of ASC speck and inflammatory responses. Nonetheless, a regulatory role by the oligomeric states of ASC specks in downstream inflammatory responses has yet to be explored, mainly due to the difficulty to produce ASC specks with varying degrees of oligomerization.

Here we demonstrate that activation of caspase-1 through the inflammasome in the extracellular space is regulated by oligomerization degrees of ASC specks. A protein binder targeting a PYD of ASC (ASC^PYD^) was developed, and structural analysis verified its inhibitory effect on the interaction between ASC^PYD^ molecules. The protein binder was shown to disassemble preformed ASC specks, generating ASC specks with low oligomeric states. ASC specks with a low degree of oligomerization were shown to recruit and process more pro-caspase-1 through facilitated homotypic interactions between CARDs of ASC (ASC^CARD^) and caspase-1(caspase-1^CARD^), resulting in an increase in the caspase-1 activation in inflammatory responses.

## Results

### Development and characterization of an ASC^PYD^-specific protein binder

**Fig. 1:**
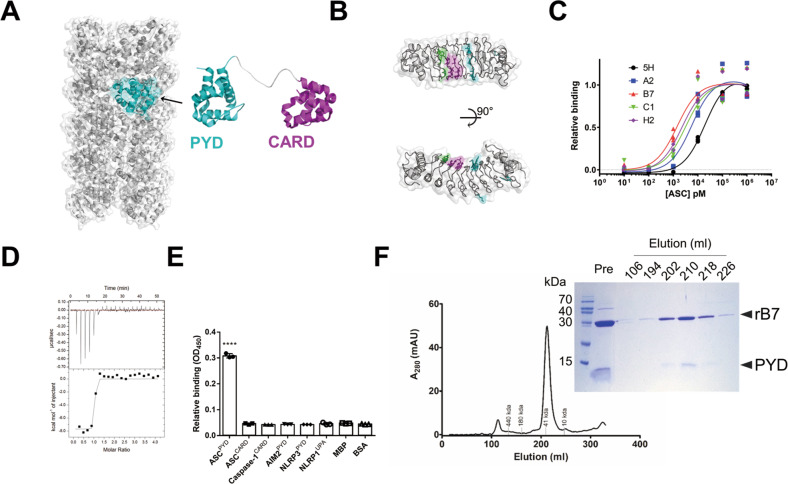
Development and characterization of an ASC^PYD^-specific protein binder. **A** Structure of ASC comprising PYD and CARD (PDB ID: 2KN6, 3J63). ASCs are oligomerized through homotypic interaction between PYDs. The surface model with gray color represents oligomerized ASC^PYD^ molecules. **B** Structural representation of the repebody scaffold (PDB ID: 3RFS) used for the development of an ASC^PYD^-specific protein binder. A repebody library was constructed by randomizing variable sites on LRR modules on a concave surface, followed by phage display selection against ASC^PYD^. Mutation sites for library construction are shown in color: first library sites in purple, second library sites in green, and additional mutation sites in cyan. **C** Relative binding affinities of an initially selected repebody (r5H) and affinity-matured repebodies against ASC^PYD^ using ELISA. The highest binding intensity was observed by rB7. The data represent the means ± SDs from triplicate experiments. **D** Titration curve of rB7 against ASC^PYD^ using ITC. The binding affinity (K_D_) of rB7 for ASC^PYD^ was determined to be 1.5 nM. Data were representative of three independent experiments. **E** Specificity of rB7. The binding of myc-tagged rB7 (4 μM) was tested against various proteins (50 nM) coated on immunoplate. BSA was used as a negative control. Relative binding was quantified by ELISA using myc-tag antibody. The data represent the means ± SDs from triplicate experiments. ^****^p < 0.0001 compared with the control (two-tailed unpaired Student’s *t-*test). **F** Elution profile of rB7 in complex with ASC^PYD^ on size-exclusion chromatography. Inset represents SDS/PAGE analysis for eluted fractions from size-exclusion chromatography.

Genetic ablation has been mainly used to control the ASC oligomerization, but such approaches are related to the assembly step of ASC specks rather than preformed ASC specks [11, 22, 23]. In addition, the oligomeric state of preformed ASC specks is difficult to control due to their complex interactions and assembly into a large size (1–2 μm) [24]. To investigate how the oligomeric states of ASC speck affect inflammatory responses, we intended to develop a protein binder that inhibits the interactions within ASC specks to produce ASC specks with a low degree of oligomerization (Fig. 1). ASC is a bipartite protein composed of CARD and PYD (Fig. 1A), and its oligomerization is mainly triggered by homotypic PYD interaction [25, 26]. The binding affinity of an ASC^PYD^ for an interacting ASC^PYD^ was estimated to be 40 to 100 μM [27]. We hypothesized that a protein binder which inhibits the interaction between ASC^PYD^ could disassemble even preformed ASC specks, generating ASC specks with low oligomeric states. ASC specks retain a densely packed oligomeric structure, and a small-sized protein binder was expected to have higher accessibility to ASC^PYD^ compared to a large-sized one such as an antibody [28]. We developed an ASC^PYD^-targeting protein binder using a non-antibody protein scaffold, termed “repebody,” which is composed of leucine-rich-repeat (LRR) modules [29]. The repebody scaffold has a horseshoe-like structure with a size of 30 kDa and high stability (Fig. 1B). Briefly, a repebody library was constructed through the randomization of variable sites on LRR modules of the concave region, followed by a phage display selection against ASC^PYD^ as described elsewhere [29]. An initial binder with a modest affinity for ASC^PYD^ was selected, followed by affinity maturation through a modular evolution process (Fig. S1). Binding affinities of an initial binder and affinity-matured ones were compared using ELISA (Fig. 1C). The most promising rB7 clone was expressed in *E. coli* and purified, and its dissociation constant (K_D_) against ASC^PYD^ was estimated to be 1.5 nM through ITC (Fig. 1D). The rB7 was observed to be highly specific for ASC^PYD^, showing a negligible binding affinity for structurally similar death-fold domains (Fig. 1E). The formation of a complex between rB7 and ASC^PYD^ was confirmed by analyzing the elution profile on size-exclusion chromatography and SDS/PAGE (Fig. 1F).

### Disassembly of recombinant ASC specks by the ASC^PYD^-specific protein binder

**Fig. 2:**
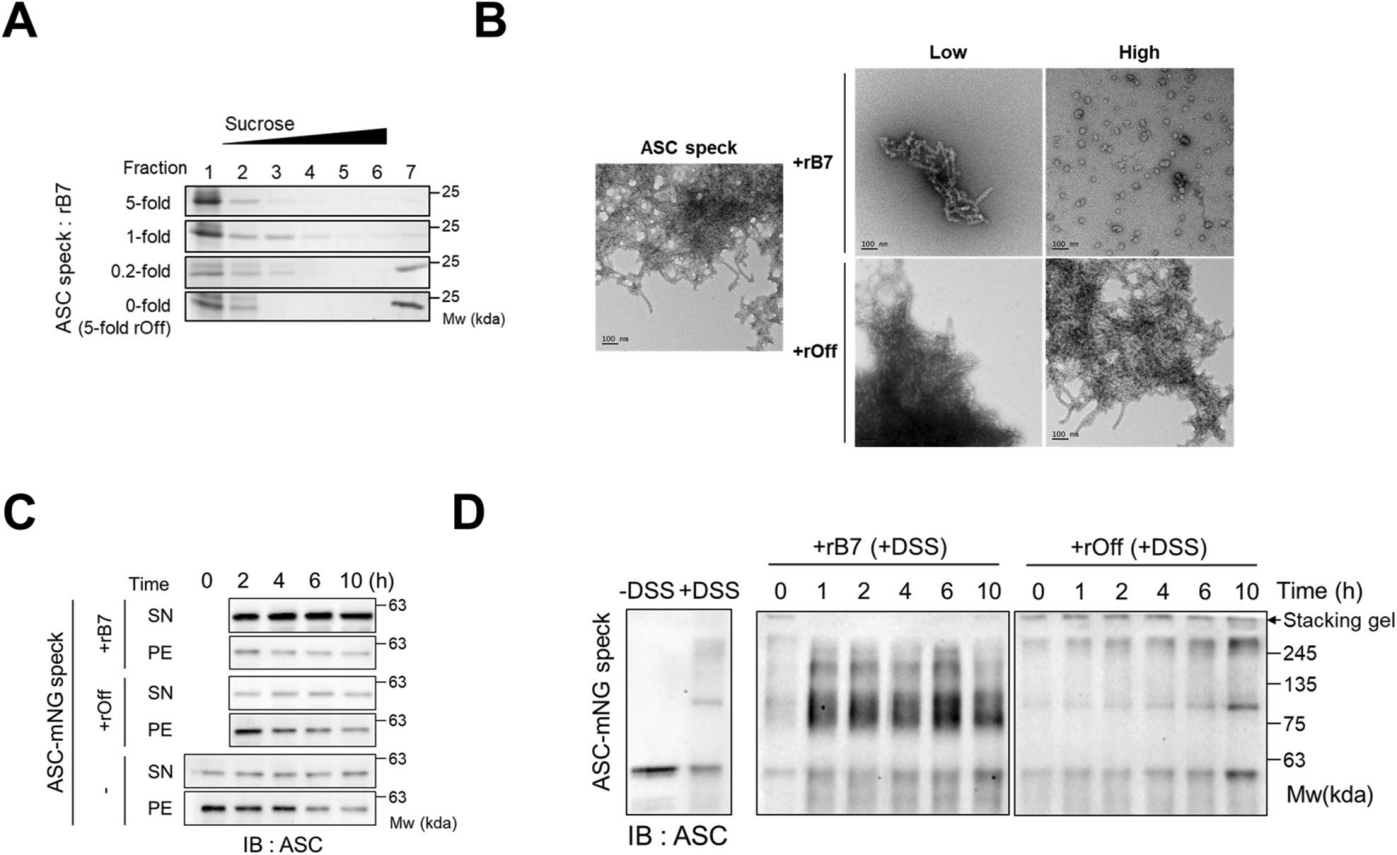
The ASC^PYD^-specific protein binder effectively disassembles preformed ASC specks. **A** The ASC levels in each fraction were obtained through sucrose-gradient (5–45%) ultracentrifugation of recombinant ASC specks. ASC specks were prepared by TEV cleavage of 5 μM MBP-fused full-length ASC, followed by the addition of rB7 as designated molar ratio for 1 h and ultracentrifugation (42,000×*g*, 10 min). rOff indicates off-target protein binder (wild-type repebody). **B** Negative stain EM images of recombinant ASC specks after treatment with rB7 or rOff. ASC specks are prepared by the same method as in (**A**). Low (15 μg/ml) and high (50 μg/ml) concentrations of rB7 or rOff were added to 30 μg/ml of preformed ASC specks for 30 min. Bars indicate 100 nm. **C** Changes in the levels of mNeonGreen-fused ASC specks in the supernatants (SN) and pellet (PE) after centrifugation (18,000×*g*, 20 min) over time. mNeonGreen-fused ASC specks were expressed and purified from HEK293 cells. ASC speck particles were diluted to ~5 × 10^5^ per μl in DPBS, followed by incubation with 10 μM of rB7 or rOff, and the relative amounts of ASC in the supernatants and pellet were analyzed through a western blot. Treatment of ASC specks with DPBS was used as the negative control. Data were representative of three independent experiments. **D**. Western blot analysis of cross-linked mNeonGreen-fused ASC specks after incubation with 10 μM of rB7 or rOff. Samples obtained at designated time intervals were cross-linked by 2 mM DSS for 30 min. Data were representative of three independent experiments.

Considering around a 10,000-fold higher affinity of rB7 for ASC^PYD^ than a homotypic interaction between ASC^PYD^ itself, we thought that rB7 could disassemble even preformed ASC specks into smaller-sized ones with low oligomeric states. To directly observe the disassembly of ASC specks by rB7, we intended to analyze the change in the oligomeric state of recombinant ASC specks in the presence and absence of rB7. For this, maltose-binding protein (MBP) was genetically fused to a full-length ASC using a linker containing a cleavage sequence of a tobacco etch virus (TEV) protease as described earlier [25], and the resulting MBP-fused ASCs were treated with a TEV protease to produce recombinant ASC specks. The resulting solution containing recombinant ASC specks was passed through a Ni-NTA column to remove the TEV protease, uncleaved MBP-fused ASC, and MBP with a polyhistidine-tag on its N-terminus. To examine the change in the oligomeric state of recombinant ASC specks by the action of rB7, the separated solution of recombinant ASC specks was incubated with different concentrations of rB7 or rOff (an off-target protein binder), followed by analysis using sucrose-gradient ultracentrifugation (Fig. 2A and Fig. S2). With the increasing concentration of rB7, the amount of high-order ASC specks in fraction 7 decreased in a concentration-dependent manner. Consequently, the portion of disassembled ASC specks was shown to increase in fractions 1–3. The addition of an excess amount of rB7 also consistently elevated the level of ASC in fractions 1–2. The action of rB7 on ASC specks was also analyzed using negative stain EM (Fig. 2b and Fig. S3). Lumpy intermediates and small-sized fragments (~50 nm) of ASC specks were observed as dominant oligomer species after 30 min incubation with rB7. It is interesting to note that small-sized fragments resembled the previously reported ring-like structure of incompletely oligomerized death domain protein [30].

We further examined the action of rB7 on ASC specks using mNeonGreen-fused full-length ASC specks that had been expressed and purified from HEK293 cells as described elsewhere [31] (Fig. S4). The solution containing mNeonGreen-ASC specks were sampled immediately after preparation in DPBS, aliquoted followed by the addition of rB7 or rOff, and the relative amounts of ASC in the pellet and supernatants were analyzed using western blot after centrifugation (18,000×*g*, 20 min) (Fig. 2C and Fig. S2). The mNeonGreen-ASC specks diluted from stock were observed to slowly disassemble only after a long incubation time. In contrast, the addition of rB7 resulted in an immediate increase in soluble ASC in the supernatants, while rOff has a negligible effect. We also investigated the disassembly of mNeonGreen-ASC specks by rB7 through chemical cross-linking using disuccinimidyl suberate (DSS) and a consequent western blot (Fig. 2D and Fig. S2). Migration of cross-linked mNeonGreen-ASC specks on SDS/PAGE were significantly reduced due to the formation of covalently bonded aggregates. Furthermore, most of the cross-linked mNeonGreen-ASCs did not move from a stacking gel to a running gel, and a marginal change was observed due to spontaneous dissociation after a long time of incubation. On the other hand, when rB7 was added, migration of small-sized fragments (70–240 kDa on cross-linking) was markedly promoted, and the amount of ASC specks with a high molecular weight (>245 kDa on cross-linking) decreased. It is noteworthy that most of the disassembled ASCs were cross-linked with rB7 (~75 kDa on cross-linking). These results indicate that the ASC^PYD^-targeting rB7 effectively broke down even preformed ASC specks into small-sized fragments through disruption of homotypic interactions between ASC^PYD^ molecules.

### Structural basis for inhibition of ASC oligomerization by rB7

**Fig. 3:**
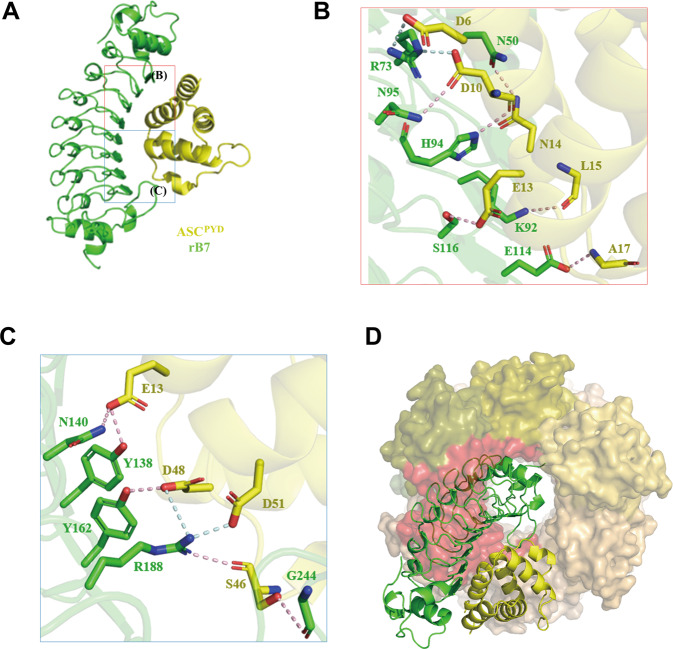
Structural basis for inhibition of ASC oligomerization by rB7. **A** One protomer structure of rB7 in complex with ASC^PYD^. rB7 and ASC^PYD^ are shown in the cartoon model. Two interaction regions are presented in detail in (**B**) and (**C**). **B** Hydrogen bonds and salt bridges are shown in dashed lines in the stick model. **C** Hydrogen bonds are represented in light pink, and salt bridges are represented in pale cyan. **D** Superimpose of rB7 in complex with ASC^PYD^ on Cryo-EM structure of ASC^PYD^ filament (PDB ID: 3J63). rB7 and ASC^PYD^ are presented in the cartoon model, and ASC^PYD^ filaments are shown in the surface model. Seven molecules of ASC^PYD^ are found nearby, and the steric clash with rB7 is highlighted in red.

To get insight into the structural basis for the inhibitory effect of rB7 on the ASC oligomerization, we determined the X-ray structure of rB7 in complex with ASC^PYD^ at 2.29 Å resolution (PDB ID: 7E5B). The crystallographic and refinement statistics are shown in Table S2. There are two protomers of ASC^PYD^ complexed with rB7 in the asymmetric unit because of crystallographic packing (Fig. S5A). The root-mean-square deviation (RMSD) value between two protomers is 0.237 Å, which implies these complexes are rigid and structurally almost identical (Fig S5B). Structural analysis revealed that ASC^PYD^ tightly interacts primarily with the concave side of rB7 through hydrogen bonds and salt bridges (Fig. 3A). Both Asp6 and Asp10 of ASC^PYD^ form salt bridges and hydrogen bonds with Arg73 of rB7, and Asn14 of ASC^PYD^ has hydrogen bonds with Asn50 of rB7. Three residues (Lys92, His94, and Asn95 of rB7) also form hydrogen bonds with Leu15 and Asp10 of ASC^PYD^, and Glu114 and Ser116 of rB7 have hydrogen bonds with Ala17 and Glu13 of ASC^PYD^, respectively (Fig. 3B). Arg188 of rB7 forms a salt bridge and hydrogen bond with Asp51 of ASC^PYD^, and it also has a salt bridge with Asp48 of ASC^PYD^. Three residues (Gly244, Tyr138, and Asn140 of rB7) form hydrogen bonds with Ser46 and Glu13 of ASC^PYD^, and both Tyr162 and Arg188 of rB7 have hydrogen bonds with Asp48 and Ser46 of ASC^PYD^, respectively (Fig. 3C). To identify crucial interaction residues between ASC^PYD^ and rB7, alanine-scanning mutagenesis was carried out for the above-mentioned residues in an MBP-fused ASC^PYD^, and relative binding to rB7 was checked through ELISA (Fig. S5C). As a result, both Glu13 and Asp48 were shown to make a major contribution to the interaction with rB7 compared to the rest of the residues.

To demonstrate the mechanism by which rB7 inhibits the ASC oligomerization, we superimposed the structure of rB7 in complex with ASC^PYD^ on the cryo-EM structure of ASC^PYD^ oligomer (PDB ID: 3J63) [25]. The cryo-EM structure of ASC^PYD^ oligomers were modeled to be composed of fifteen ASC molecules, and charge interactions play a major role in the formation of ASC oligomers. As a result, seven ASC molecules are found nearby, causing a steric clash with rB7 (Fig. 3D). The cryo-EM structure revealed that 27 residues of ASC^PYD^ are involved in the oligomerization of ASC. Among them, eight residues are shown to interact with rB7 at the same time, and five residues are involved in ASC^PYD^ oligomerization (Fig. S5D). Taken together, rB7 is likely to effectively inhibit ASC oligomerization by binding to the interface of ASC^PYD^ oligomers, generating fragmented ASC specks with a low degree of oligomerization.

### Enhanced interaction between ASC^CARD^ and caspase-1^CARD^ by disassembled ASC specks

**Fig. 4:**
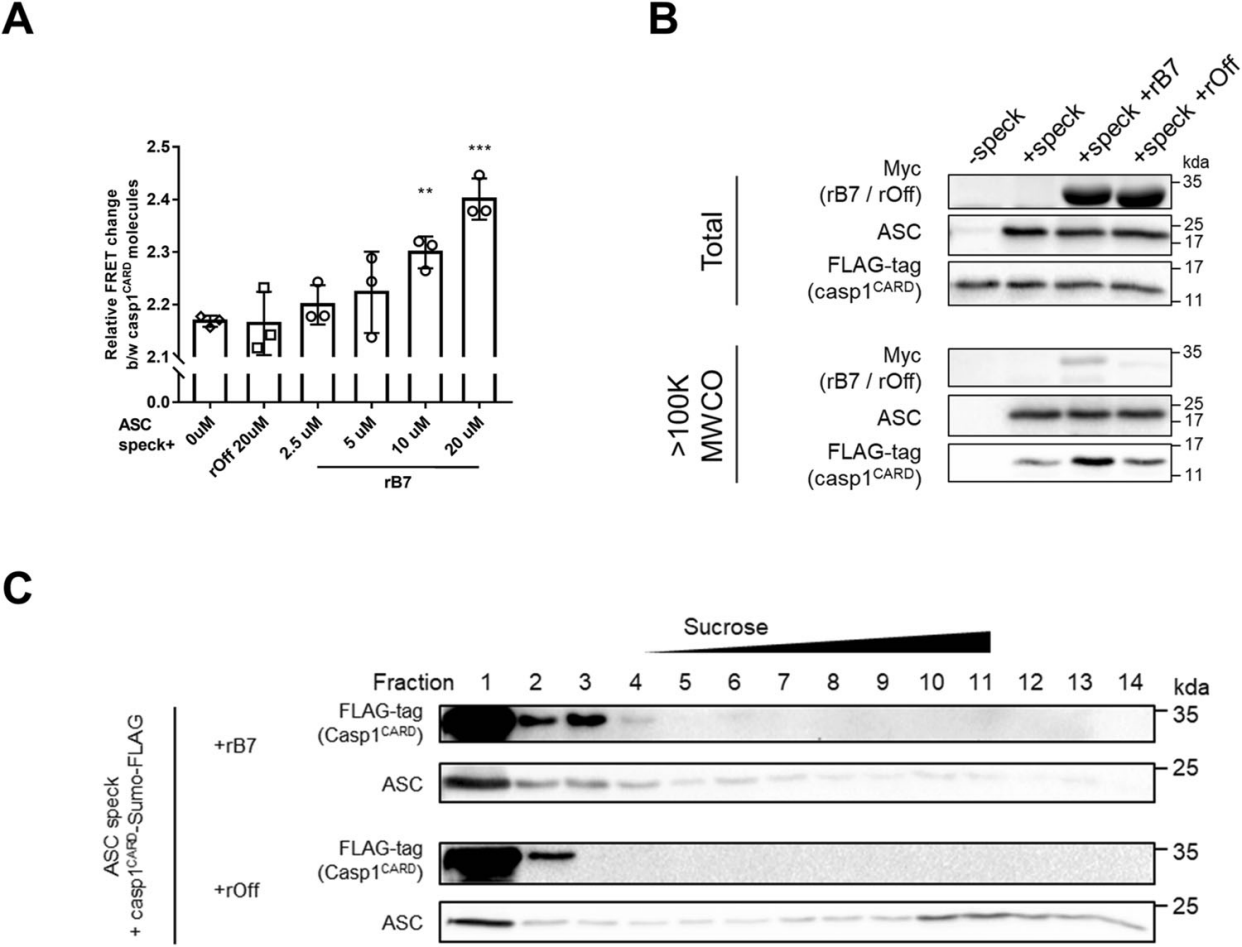
Enhanced interactions between ASC^CARD^ and caspase-1^CARD^ by rB7-treated ASC specks. **A** Changes in FRET efficiency by the clustering of caspase-1^CARD^ molecules in the presence of recombinant ASC specks which had been treated with different rB7 concentrations. MBP-fused caspase-1^CARD^-SUMO molecules labeled with DyLight 550 and DyLight 650 were mixed with TEV protease in the presence of recombinant ASC specks which had been treated with rB7 or rOff. ASC specks were prepared using the same method as in Fig. 2A. FRET efficiency after 45 min was measured and normalized by the initial value. The data represent the means ± SDs from triplicate experiments. ^***^p < 0.001 and ^**^p < 0.01 compared with the control (two-tailed unpaired Student’s *t*-test). **B** The level of clustered caspase-1^CARD^ by rB7-treated recombinant ASC specks after filtration (100 K MWCO). ASC specks prepared from 2.5 μM MBP-fused full-length ASC were treated with 5 μM rB7 or rOff for 1 h, followed by 1 h incubation with 10 μM MBP-fused FLAG-tagged caspase-1^CARD^ and TEV protease. Clustered caspase-1^CARD^ by ASC specks was enriched by filtration (100 K MWCO) and analyzed through western blot. Data were representative of three independent experiments. **C** Analysis of tethered caspase-1^CARD^ and ASC in each fraction obtained through sucrose-gradient (5–45%) ultracentrifugation of recombinant ASC specks. ASC specks were prepared using the same method as in (**A**) and treated with 5 μM of rB7 or rOff for 30 min, followed by incubation with 15 μM MBP-fused caspase-1^CARD^-SUMO-FLAG and TEV protease for 1 h. Data were representative of three independent experiments.

ASC specks are known to act as a platform for recruiting and processing premature caspase-1 via interactions between ASC^CARD^ and caspase-1^CARD^ when cells are stimulated [9, 32, 33]. To check a correlation between the oligomeric states of ASC specks and inflammatory responses, we investigated the recruitment of caspase-1^CARD^ by ASC^CARD^ by measuring the FRET efficiency between caspase-1^CARD^ through clustering of caspase-1^CARD^. MBP was fused to caspase-1^CARD^-SUMO using a linker containing TEV protease recognition sequence as previously described [25], and the resulting MBP-fused caspase-1^CARD^-SUMO was labeled with respective maleimide-activated FRET pair probes (DyLight^TM^ 550 and DyLight^TM^ 650, respectively) using free cysteine residues [34]. MBP-fused caspase-1^CARD^-SUMO molecules that had been labeled with each probe were mixed together, followed by the addition of TEV protease in the presence of recombinant ASC specks, which had been incubated with rB7 or rOff. As shown in Fig. 4A, the FRET efficiency between caspase-1^CARD^-SUMO molecules increased in the presence of ASC specks, showing a dependence on the rB7 concentration compared to the controls. ASC specks are amorphous aggregates comprising ASC^PYD^ filaments that are linked through homotypic interaction between ASC^CARD^ [28]. Based on the results, it is likely that disassembled ASC specks with low oligomeric states had more solvent-exposed ASC^CARD^, facilitating the recruitment and clustering of caspase-1^CARD^ through interactions between ASC^CARD^ and caspase-1^CARD^ and, consequently, FRET efficiency between caspase-1^CARD^ molecules increased.

We further analyzed the relative quantity of caspase-1^CARD^ tethered to ASC specks using molecular weight cut-off (MWCO) filtration. Recombinant ASC specks were incubated with rB7 or rOff, and FLAG-tagged caspase-1^CARD^ molecules were added. After 1 hr of incubation, the resulting solution was filtrated using a 100 K MWCO centrifugal filter to enrich caspase-1^CARD^ molecules tethered to ASC specks, excluding free caspase-1^CARD^ (Fig. 4B and Fig. S6). In the absence of recombinant ASC specks, the remaining caspase-1^CARD^ molecules after filtration were barely observed, which indicates that caspase-1^CARD^ itself does not easily self-polymerize due to low affinity, as reported elsewhere [35]. However, when recombinant ASC specks were added, we detected the caspase-1^CARD^ molecules that were tethered to ASC specks and did not pass through the filter. The amount of tethered caspase-1^CARD^ molecules was shown to increase when rB7-treated ASC specks were added. This result implies that the disassembly of ASC specks by rB7 resulted in an increase in the level of tethered caspase-1^CARD^ molecules through enhanced interactions between ASC^CARD^ and caspase-1^CARD^.

We next examined a linkage between the oligomeric states of ASC specks and recruiting of caspase-1^CARD^ using sucrose-gradient ultracentrifugation. For this, the recombinant ASC specks which had been treated with rB7 or rOff were incubated with caspase-1^CARD^-SUMO-FLAG for 1 hr, followed by separation into fourteen fractions by sucrose-gradient ultracentrifugation, and the levels of ASC and tethered caspase-1^CARD^-SUMO-FLAG in each fraction were analyzed (Fig. 4C and Fig. S6). In sucrose-gradient ultracentrifugation, the degree of oligomerization of ASC speck increases with the increasing fraction number, and fraction 1 was considered to mainly contain monomeric ASC. It is well known that oligomerized ASC recruits premature caspase-1 and ASC monomers cannot cluster premature caspase-1 [25, 36]. When ASC specks were treated with rOff, ASC was mainly detected in fraction 1 and fractions 10–14, and the level of caspase-1^CARD^-SUMO-FLAG that had co-migrated with highly oligomerized ASC specks was shown to be very low and detected only in fraction 2. In contrast, treatment of ASC specks with rB7 resulted in a drastic decrease in the level of ASC in fractions 10–14. At the same time, the ASC levels in fractions 2–4, which contain low oligomeric states of ASC specks, were observed to increase, and a significant amount of caspase-1^CARD^-SUMO-FLAG accumulated in the same fractions. These results support that rB7-treated ASC specks recruited more caspase-1^CARD^ molecules through facilitated homotypic interactions between ASC^CARD^ and caspase-1^CARD^ than non-treated ASC specks.

### Linkage between the processing of premature caspase-1 and oligomeric states of extracellular ASC specks

**Fig. 5:**
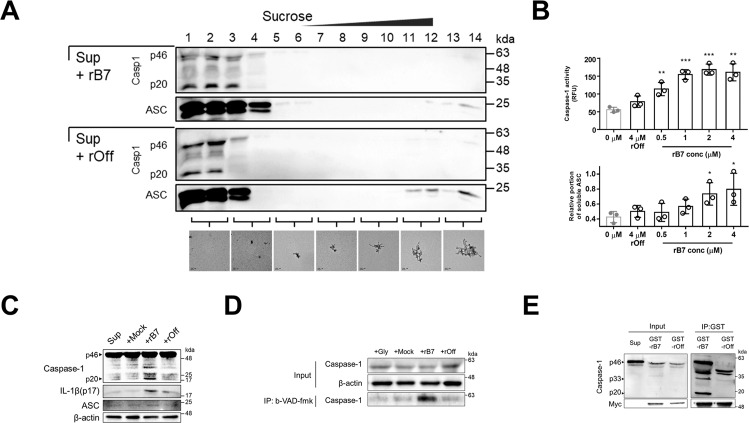
Linkage between the processing of premature caspase-1 and oligomeric states of extracellular ASC specks. **A** Analysis of tethered caspase-1 and ASC in each fraction obtained through sucrose-gradient (5–45%) ultracentrifugation of endogenous ASC specks. Cell-free supernatants from stimulated THP-1 cells containing ASC specks were incubated with 5 μM of rB7 or rOff for 1 h, followed by sucrose-gradient ultracentrifugation (167,000×*g*, 10 min), and the levels of caspase-1 (Casp1) and ASC in each fraction were analyzed using western blot (top panel). Negative stain EM images of ASC specks in each fraction were obtained through sucrose-gradient ultracentrifugation (bottom panel) at the same condition as in the top panel except for the use of recombinant ASC specks. The bars indicate 100 nm. Data were representative of three independent experiments. **B**. Caspase-1 activation through extracellular ASC specks in the presence of varying concentrations of rB7. After various concentrations of rB7 or rOff were added to the cell media, LPS/nigericin was used to stimulate THP-1. A fluorogenic substrate (z-YVAD-AFC) was added immediately after stimulation, and caspase-1 activity was assayed by measuring the change in the fluorescence intensity. Soluble ASC in the cell-free supernatants was separated through centrifugation (18,000×*g*, 10 min), and the amounts of ASC were determined by band intensity on western blot. The data represent the means ± SDs from triplicate experiments. ^***^p < 0.001, ^**^p < 0.01, and ^*^p < 0.05 compared with the control (two-tailed unpaired Student’s *t*-test). **C** Immunoblot analysis of processed caspase-1 species and mature IL-1β when cell-free supernatants from stimulated THP-1 cells were incubated with 5 μM of rB7, rOff, or DPBS (Mock) for 1 h. Data were representative of three independent experiments. **D** Immunoblot analysis of active caspase-1 labeled with biotin-VAD-fmk in the presence of rB7. 3 μM of rB7 or rOff were added to the extracellular space of THP-1 cells as in (**B**). Biotin-VAD-fmk was added 30 min before stimulation with nigericin. The labeled caspase-1 was pulled down using streptavidin-conjugated dynabead. The eluted fraction was analyzed using a western blot. “Gly” indicates the addition of glycine before stimulation to prevent pyroptotic cell lysis. Data were representative of three independent experiments. **E** Immunoblot of caspase-1 eluted from co-immunoprecipitation using GST-fused rB7 or rOff in stimulated cell-free supernatants. Myc-tag was fused to the C-terminus of GST-fused rB7 and rOff for detection. Data were representative of three independent experiments.

Based on our observation that rB7-treated ASC specks recruited more caspase-1^CARD^ molecules than non-treated ASC specks, we intended to check if such rB7-treated ASC specks eventually lead to an increase in inflammatory response even after pyroptosis using a model cell line. ASC specks with different sizes are known to be formed through an inflammatory activation process in the intracellular space [17–19, 37, 38], and are released outside the cell after pyroptotic cell lysis, continuing to activate pro-caspase-1 in the extracellular space. The oligomeric state of ASC speck varies depending on surrounding factors, and a similar result was observed in this study (Fig. S7). We first examined the interaction between caspase-1 and endogenous ASC specks with varying oligomeric states through ultracentrifugation. The cell-free supernatants containing extracellular ASC specks were obtained by stimulating PMA (phorbol 12-myristate 13-acetate)-differentiated THP-1 cells using LPS/nigericin which is a known activator for NLRP3 inflammasome. The NLRP3 inflammasome has been the most widely used model for studying inflammasome formation and pyroptosis. In addition, stimulation with nigericin is suitable for studying extracellular ASC specks, while excluding uptake by bystander cells, because most of the cells simultaneously undergo pyroptotic cell death in a short time [19, 39, 40]. The cell-free supernatant was incubated with 5 μM of rB7 for 1 hr, followed by separation into fourteen fractions through sucrose-gradient ultracentrifugation, and the levels of ASC and caspase-1 species in each fraction were analyzed by western blot (Fig. 5A and Fig. S8). The size of endogenous ASC specks in each fraction was estimated by comparison with recombinant ASC specks under the same ultracentrifugation condition using negative stain EM (Fig. 5A, bottom). The cell-free supernatants from stimulated THP-1 cells contained large-sized ASC specks as well as nearly monomeric ASC, which is consistent with previous reports [41]. Fractions 1 and 2 were shown to contain nearly monomeric ASC and caspase-1, whereas ASC oligomers with an average size of 30–50 nm were found in fractions 3 and 4. ASC specks were observed to migrate towards the bottom of the sucrose cushion, and the level of ASC oligomers in fractions 3 and 4 increased when ASC specks were treated with rB7. Consistent with the experimental results using recombinant caspase-1^CARD^ and ASC specks, only a faint band of caspase-1 was observed in fractions 11–14, which contained ASC specks with a high degree of oligomerization. In contrast, a significant amount of caspase-1 species was shown to cluster together with small-sized ASC specks, which had been produced by the action of rB7. As expected, processed caspase-1 (p20) also increased as the clustering of caspase-1 enhanced. Taken together, small-sized ASC specks produced by rB7 seem to recruit more premature caspase-1 than non-treated ASC specks, accelerating the caspase-1 activation.

To further examine the modulation of the caspase-1 activation by oligomeric states of ASC specks, different concentrations of rB7 were added to the culture medium of THP-1 cells, and the caspase-1 activity was measured using a fluorogenic substrate (z-YVAD-AFC) immediately after stimulation (Fig. 5B). Relative amounts of soluble ASC in the extracellular space were also quantified after centrifugation (18,000×*g*, 10 min) of cell-free supernatants. Unlike small molecule compounds, rB7 cannot penetrate into the cells, acting only on extracellular ASC specks, and produces ASC specks with varying degrees of oligomerization (Fig. S9). It is noteworthy that rB7 had a negligible effect on the pyroptotic cell death rate after stimulation, the total amount of ASC released from THP-1 cells and the extracellular level of β-actin (Figs. S10, S11). The caspase-1 activity was shown to increase as the level of soluble ASC in the supernatants increased by the action of increasing rB7 concentration, reaching a plateau. This result also supports that disassembled ASC specks triggered more effectively the caspase-1 activation in the extracellular space than highly dense ASC specks.

We next analyzed the processing of premature caspase-1 when cell-free supernatants separated from the stimulated THP-1 cells were incubated with rB7 (Fig. 5C and Fig. S8). The levels of processed caspase-1 species, including p20 and the intermediate species, were observed to increase by rB7, and this seems to be due to enhanced processing of premature caspase-1 by rB7-treated ASC specks. Accordingly, the level of mature IL-1β also increased with the increasing caspase-1 activity. We further conducted a pull-down assay using biotin-VAD-fmk, which specifically binds to the active caspase-1 (Fig. 5D and Fig. S8). The amount of active caspase-1 significantly increased when THP-1 cells were stimulated with LPS/nigericin in the presence of rB7. In the end, we intended to check whether active caspase-1 is directly linked to rB7-bound ASC specks. Caspase-1 species, which are in complex with the rB7-bound ASC specks, were analyzed through co-immunoprecipitation (Co-IP) using glutathione-*S*-transferase (GST)-fused rB7. As shown in Fig. 5E and Fig. S8, the processed caspase-1 species, including p20 were eluted in the highly enriched form in the rB7-bound fractions, which supports the interactions among the rB7, ASC specks, and caspase-1 species. Based on the results, increased caspase-1 activity is likely to be driven by facilitated recruiting and processing of premature caspase-1 through enhanced interaction between caspase-1^CARD^ and ASC^CARD^ of disassembled ASC specks.

## Discussion

**Fig. 6:**
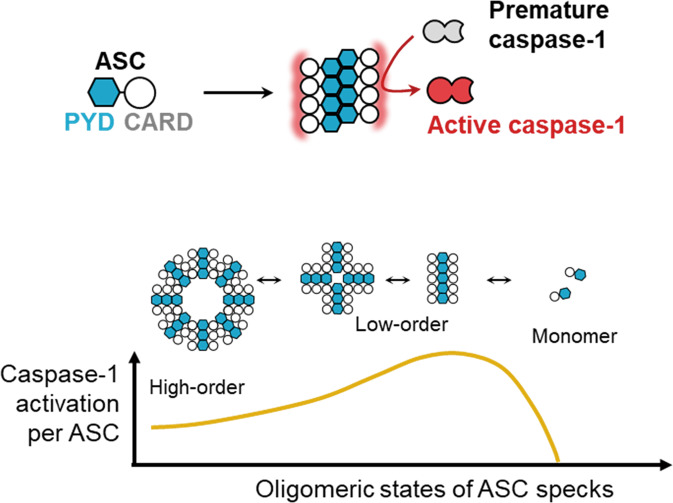
Proposed model for enhanced activation of caspase-1 in the extracellular space through ASC specks with low oligomeric states. ASC specks with a low degree of oligomerization have more ASC^CARD^ available for the homotypic interactions between ASC^CARD^ and caspase-1^CARD^, increasing the activation of caspase-1 in inflammatory responses.

A linkage between oligomeric states of ASC specks and inflammatory responses was largely unexplored, mainly due to the difficulty to produce ASC specks with varying degrees of oligomerization. With the ASC^PYD^-specific protein binder (rB7), we were able to investigate how the oligomeric states of ASC specks affect caspase-1 activation through inflammasomes. Structural analysis verified that rB7 effectively inhibits the ASC oligomerization by binding to the interface between ASC^PYD^. Treatment of ASC specks with rB7 was shown to significantly enhance the caspase-1 activation through increased homotypic interactions between ASC^CARD^ and caspase-1^CARD^. Based on the results, we proposed a model for a regulatory role by oligomeric states of ASC specks on the activation of inflammatory caspase-1 (Fig. 6). The activation of inflammatory caspase-1 is known to be primarily initiated by the formation of ASC specks [15, 16, 20]. Nonetheless, fragmented ASC specks with a low oligomeric state are likely to facilitate the caspase-1 activation in the extracellular space through enhanced recruitment of pro-caspase-1. It is interesting to note that other oligomer-forming proteins, such as heat-shock proteins and amyloids, also favor small-sized oligomers for higher activity [42–44].

The structural stability of ASC specks is affected by the orchestration of sensing proteins, osmolality, ion-flux, and antagonizing proteins such as PYD-only proteins (POPs) [9, 11, 19]. Considering that the size of ASC specks are varied by the above factors, the disassembly of ASC specks is thought to play a role in balancing the inflammatory cascade. It is conceivable that the oligomerization nature of ASC has a role in reducing the over-activation of inflammatory responses after pyroptosis, and at the same time, it regulates the appropriate activation of inflammatory responses. Understanding the immunological consequences of densely packed and disassembled ASC specks, which are possibly affected by various factors such as microenvironments, the lineage of immune cells, and the type of inflammasomes, can provide insight into controlling the inflammasome-mediated inflammatory process as well as the development of inflammasome-targeting drugs. Various chronic inflammatory diseases are known to be caused by the dysregulated activation of inflammasomes, but inflammasome signaling also plays a crucial protective role in the anticancer responses, antiviral responses, and clearance of pathogens [9, 45, 46]. In this regard, bidirectional regulation of inflammasome activation might offer a beneficial therapeutic approach. For example, fragmentation of ASC speck can be used to boost the immunosurveillance function by the inflammasome, which will trigger the immediate immunological response against viruses and other pathogens. However, considering some controversy over the beneficial role of inflammasome signaling depending on diseases, further study is necessary to explore whether modulation of the inflammasome response is advantageous in human pathological conditions.

## Materials and methods

### Reagents

Ultrapure LPS from Escherichia coli strain O111:B4 was purchased from Merck (#L3024), and nigericin was received from Invitrogen. Anti-ASC mouse monoclonal antibody (1:1000 dilution, sc-514414) and anti-c-Myc mouse monoclonal antibody (1:1000 dilution, sc-40) were from Santa Cruz, respectively. Rabbit polyclonal antibody against Caspase-1 (1:1000 dilution, 2225) was received from Cell Signaling Technology. Anti-β-actin mouse monoclonal antibody (1:10,000 dilution, A5316) was received from Sigma-Aldrich. HRP-conjugated antibodies against mouse and rabbit IgG were obtained from Biorad. Cell counting kit-8 (CCK-8) for a cell viability assay were obtained from Dojindo. DSS (A39267) for the cross-linking of ASC was purchased from Pierce. The gene constructs were synthesized from IDT Technologies. LB broth and antibiotics were received from Duchefa (Netherlands). Origami B (DE3) competent cells for protein expression were purchased from Merck Biosciences (Germany), and isopropyl β‐D‐1‐thiogalactopyranoside (IPTG) was obtained from LPS Solution (Korea). The Ni-NTA agarose resin for affinity purification was from Qiagen.

### Protein expression and purification

Full-length ASC, caspase-1^CARD^-SUMO, and ASC^PYD^ were fused to the C-terminal of MBP with a linker possessing a recognition sequence of TEV protease. The resulting gene constructs were cloned into a pET21a vector, and a hexahistidine tag was introduced at the N-terminus of MBP for affinity purification. The vector harboring the gene constructs was transformed into BL21 (DE3) cells, and the resulting cells were cultured in an LB medium overnight until the optical density at 600 nm reached 0.4–0.8. Cells were treated with 0.2 mM IPTG and further grown overnight at 18 °C. Harvested cells were disrupted by sonication, and a cell-free extract was subjected to affinity purification followed by size-exclusion chromatography. An ASC^PYD^-specific repebody was cloned into a pET21a vector with a myc tag for detection and a six-histidine tag for affinity purification at the C-terminus. The constructed gene was transformed into *E. coli* strain Origami B cells, and the cells were cultured in an LB medium followed by induction with 0.5 mM IPTG. Cells were further grown at 16 °C overnight and harvested through centrifugation. The collected cells were disrupted through sonication, and a cell-free extract was subjected to affinity purification using Ni-NTA agarose and size-exclusion chromatography. The LPS was removed through a phase separation using Triton X-114 (Sigma-Aldrich), as described elsewhere [47, 48]. All downstream experiments using repebody and MBP-fused constructs, respectively, used DPBS (pH 7.4) and 20 mM HEPES (pH 7.2) buffer containing 150 mM NaCl, and 1 mM TCEP by default.

For structural determination, rB7 was expressed and purified in the absence of a tag, and ten residues from the C-terminus of ASC ^PYD^ were truncated for more favorable crystal packing. The cells expressing rB7 were cultured in an LB medium, harvested, and disrupted as described above. A cell-free extract was incubated at 60 °C for 30 min, and the soluble fraction was recovered through centrifugation at 18,000×*g* for 1 h, followed by filtration using a 0.22-μm syringe filter and purification using size-exclusion chromatography (HiLoad 16/600 Superdex 75 pg, Cytiva).

### Isothermal titration calorimetry (ITC)

The binding affinity of rB7 for ASC^PYD^ was measured using ITC (MicroCal-iTC 200, Malvern Instruments) in 20 mM HEPES (pH 7.2) buffer containing 150 mM NaCl, and 1 mM TCEP at 25 °C. Briefly, 0.4 mM of rB7 in a syringe was injected into 0.02 mM MBP-fused ASC^PYD^ in a cell. The ITC measurements were conducted for a total 20 injections and stirred at 1000 rpm. The initial injection was excluded for data analysis. The dissociation constant was determined using Origin (OriginLab).

### Selection of an ASC^PYD^-specific protein binder and affinity maturation

An ASC^PYD^-specific repebody was selected using a phage display as described previously [49]. Briefly, a repebody library was constructed by randomizing six variable sites on LRRV2 and LRRV3 modules using overlap PCR with degenerate codon followed by insertion into a phagemid pBEL118N vector derived from pTV118N. The resulting phagemid was transformed into an *E.coli* strain TG-1 through electroporation, and the phage-displayed library was subjected to bio-panning against MBP-fused ASC^PYD^. The target protein was coated on an immunotube, followed by treatment with 2% BSA. The initial clones were selected through five rounds of a bio-panning procedure, and the clone with the highest binding affinity was selected using phage ELISA. The selected clone was subjected to affinity maturation through a modular evolution approach. Four additional residues on the LRRV1 module were randomized and inserted into a phagemid pBEL118N vector. The constructed library was phage-displayed and subjected to a bio-panning against the target. The clone with the highest binding affinity for the target was selected using phage ELISA.

### Comparison of relative binding by ELISA

MBP-fused ASC ^PYD^ variants (D6A, E10A, E13A, E48A, D51A, and D10A/E13A/D48A) were introduced into the wild-type of MBP-fused ASC^PYD^ construct using PCR-based site-directed mutagenesis. ASC^CARD^, caspase-1^CARD^, AIM2^PYD^, NLRP3^PYD^, and NLRP1^UPA^ were fused to MBP and purified similarly to MBP-fused ASC^PYD^. Proteins were coated on immunoplate overnight at 4 °C, followed by treatment with a blocking solution containing 1% BSA and 0.1% Tween 20 in PBS (pH 7.4). A designated concentration of myc-tagged rB7 was treated for 2 h, and a relative binding was quantified using myc-tag antibody. The plate was rinsed with a washing solution containing 0.1% Tween 20 in PBS between every step.

### Crystallization and structure determination of rB7 in complex with ASC^PYD^

The complex protein between rB7 and ASC^PYD^ was concentrated up to 6.4 mg/ml and used for crystallization. Initial crystallization screening was conducted by using a Mosquito robot (TTP Labtech), and several small and thin crystals appeared at 0.2 M ammonium sulfate, 0.1 M Bis-Tris: HCl pH 5.5, 25% (w/v) PEG 3350. Additive screening ^TM^ (Hampton Research) was used to improve crystals, and the appropriate size of single crystals appeared at condition 47 (0.1 M TCEP hydrochloride). The complex crystals were quickly soaked into a crystal buffer containing 25% glycerol to protect the crystals from the low temperature of the liquid nitrogen. X-ray diffraction of the complex crystal was conducted to collect diffraction images using a 5C beamline at the Pohang Accelerator Laboratory (PAL, South Korea). Integration of the images was conducted using HKL2000, and scaling of the mtz file was also performed using the CCP4 program [50]. The complex crystal belongs to the space group P4_3_2_1_2 with a = 79.48 Å, b = 79.48 Å, and c = 279.20 Å in a cell unit. The initial phase was obtained through a molecular replacement (MR) by the PHENIX Phaser program using the ASC^PYD^ structure (PDB ID: 3J63) and the repebody structure (PDB ID: 6LBX) as the initial model [25, 51]. Model building was conducted using the Coot program, and refinement was carried out using the PHENIX Refine program [52]. The three-dimensional model of the complex was visualized using PyMOL (Version 2.3.3, Schrödinger, LLC).

### Preparation of recombinant ASC specks in HEK293 cells

mNeonGreen-fused ASC specks were produced and purified as described elsewhere [31]. Briefly, a full-length ASC was genetically fused to mNeonGreen using a linker, and the resulting construct was inserted into pcDNA3.1 and transfected to HEK293 cells using Lipofectamine LTX plus (Invitrogen). HEK293 cells transiently expressing ASC-mNeonGreen were harvested and resuspended in a 20 mM HEPES-KOH buffer (pH 7.5) containing 320 mM sucrose, 10 mM KCl, 1.5 mM MgCl_2_, 1 mM EDTA, and 1 mM EGTA. Cells were lysed by repeatedly passing through a syringe needle and freeze/thawing. The supernatant was recovered through centrifugation at 400×*g* for 8 min and diluted with a CHAPS buffer (pH 7.5) containing 20 mM HEPES-KOH, 5 mM MgCl_2_, 0.5 mM EGTA, 0.1 mM PMSF, and 0.1% CHAPS. The solution was filtered through a 5-μm centrifugal filter (Millipore), and the resulting filtrate was diluted with 1 volume of a CHAPS buffer followed by centrifugation. Using 40% Percoll layer centrifugation of the pellet resuspended with a CHAPS buffer, mNeonGreen-fused ASC specks were obtained as fluorescent particles (Fig S4). The purified mNeonGreen-fused ASC specks were loaded on a disposable hemocytometer, and the number of particles per μl were manually counted by a fluorescence microscope. Cross-linking was conducted through the addition of 2 mM DSS for 30 min. Cross-linked ASC was analyzed using a western blot by applying the same procedure as described above.

### Preparation and negative stain EM imaging of ASC specks

ASC specks were prepared through an expression of MBP-fused ASC in *E. coli* according to the method with a slight modification [25, 26]. Briefly, 0.25 mg/ml of MBP-fused ASC was cleaved with 0.06 mg/ml of a TEV protease (Enzynomics, Korea) for 30 min, and the reaction mixture was incubated with Ni-NTA agarose resin for 1 h with shaking to remove the MBP and TEV protease. The ASC specks formed were separated using a disposable column (Pierce). Carbon-covered Cu grids (1GC400, Graticules Optics) were glow discharged for 20 s under 30 mA using a glow discharge cleaning system (PELCO easiGlow^TM^). A solution containing ASC specks was applied to the grid and incubated for 1 min at room temperature. To check the dissociation of ASC specks through the action of an ASC^PYD^-specific rB7, ASC specks were incubated with rB7 for a predetermined time, and the resulting solution was loaded onto the grid. Following the blotting with filter paper, the grid was washed twice with distilled water and 1.5% (w/v) uranyl acetate. The grid was further incubated in the uranyl acetate droplet for 1 min, followed by blotting with filter paper. The resulting grid was air-dried and imaged using an electron microscope (F20 Tecnai) equipped with a CCD camera (Gatan).

### Western blot

Prepared samples were loaded onto a 15 or 4–15% (for the cross-linked protein) acrylamide gel, respectively. The proteins on the gel were transferred onto a nitrocellulose membrane (GE Healthcare), followed by treatment with a blocking solution containing 5% BSA and 0.1% Tween 20 in PBS (pH 7.4), and incubated with a blotting buffer containing a primary antibody at 4 °C overnight. The membrane was washed with PBS containing 0.1% Tween 20 and incubated with an appropriate HRP-conjugated secondary antibody. The membrane was treated with a western Chemiluminescent HRP substrate (Merck) and analyzed using a Chemidoc system (Biorad).

### Fractionation through sucrose-gradient ultracentrifugation

A sucrose-gradient was manually constructed by layering DPBS (pH 7.4) containing 45 to 5% sucrose in an ultra-clear centrifuge tube (Beckman Coulter). The cell-free supernatants or solution containing recombinant ASC specks was loaded on top of the gradient, followed by centrifugation for 10 min at 4 °C (Optima XE-100 SW55Ti rotor, Beckman Coulter). The rotor speed was set to 32,000 rpm and 8000 rpm for endogenous and recombinant ASC specks, respectively. Prior to ultracentrifugation, an ASC^PYD^-targeting rB7 or rOff was added to the cell-free supernatants and incubated for 1 h. The gradient was divided into a designated number of fractions, and each fraction was analyzed through a western blot.

### Complex formation between rB7 and ASC^PYD^

MBP-fused ASC^PYD^ and rB7 were mixed at a 1:1 molar ratio and incubated for 1 h at 4°C. The mixture was applied to the size-exclusion column (HiLoad 16/600 Superdex 200 pg, Cytiva) with 20 mM Tris-HCl (pH 8.0), 500 mM NaCl, and 1 mM DTT. The complex between rB7 and MBP-fused ASC^PYD^ was collected and treated with TEV protease to separate MBP and rB7 in complex with ASC^PYD^ overnight at 4 °C. To remove MBP, amylose resin (New England Biolabs) was used for 1 h at 4°C. Finally, flow-through was applied to the size-exclusion chromatography (HiLoad 16/600 Superdex 200 pg, Cytiva) with 20 mM Tris-HCl (pH 8.0), 500 mM NaCl, and 1 mM DTT.

### Dye labeling and FRET assay

Dye labeling and FRET assay were carried out according to the previously described method with a slight modification [34]. MBP-fused caspase-1^CARD^ was mixed with maleimide-activated FRET pair probes (DyLight^TM^ 550 and DyLight^TM^ 650) in 20 mM HEPES buffer (pH 7.2) containing 500 mM NaCl and 1 mM TCEP, and incubated overnight at 4 °C without shaking. The excess dyes were quenched by β-mercaptoethanol, and labeled MBP-fused caspase-1^CARD^ was immobilized on amylose resin (NEB), followed by washing with 20 mM HEPES buffer containing 750 mM NaCl, 3 mM β-mercaptoethanol, and 2% glycerol. The resulting amylose resin was resuspended in the same buffer and mixed with 5 μM ASC specks, followed by the addition of TEV protease for the release of caspase-1^CARD^. FRET efficiency was determined by measuring the emission intensities at 578 and 678 nm, respectively, with the excitation at 522 nm.

### Cell-free supernatants containing endogenous ASC specks

Cell-free supernatant was obtained from stimulated THP-1 cells (Korean Cell Line Bank). THP-1 was maintained in RPMI-1640 supplemented with 10% FBS and 1% penicillin-streptomycin (Capricorn Scientific). Approximately 1 × 10^7^ cells/ml were seeded in a 48-well plate and incubated with 100 nM PMA for ~48 h in an RPMI-1640 medium for differentiation. Following the washing with DPBS, the medium was changed to fresh serum-free RPMI-1640. The cells were primarily stimulated with 1 μg/ml of LPS for 2 h, and subsequently, NLRP3 activation was achieved using 10 μM nigericin for 1 h. Following stimulation, the cell-free supernatants were collected through centrifugation (600×*g*, 5 min).

### Assay of caspase-1 activity

Approximately 5 × 10^5^ cells/ml were seeded in a 48-well plate, and THP-1 cells were differentiated as described earlier. NLRP3 activation was achieved by stimulation using LPS/nigericin. The addition of rB7 or rOff at the designated concentrations was carried out for 2 h prior to LPS stimulation. Following stimulation, the cell-free supernatants were collected through centrifugation (600×*g*, 5 min), and caspase-1 activity was assayed using a fluorogenic substrate z-YVAD-AFC (Thermo Scientific).

### Co-immunoprecipitation

For co-immunoprecipitation of a GST-fused rB7, GST was genetically fused to rB7 using a pGEX4T-1 vector. The expression vector was transformed into *E. coli*, and a GST-fused rB7 was expressed using the same procedures as described above. The cells were harvested and disrupted through sonication, and a cell-free extract was obtained using centrifugation. The soluble fraction containing a GST-fused rB7 was incubated with GST resin for 4 h at 4 °C, and the resin was washed with DPBS containing 0.05% Tween 20, followed by blocking with DPBS containing 0.05% Tween 20 and 2% BSA at room temperature for 1 h. After washing twice with DPBS containing 0.05% Tween 20, the resulting resins were incubated with cell-free supernatants from the stimulated THP-1 cells. A protease and phosphatase inhibitor cocktail were added. The resins and cell-free supernatants were mixed and incubated for 30 min at room temperature with gentle shaking. The resins were washed twice with a DPBS containing 0.05% Tween 20 and filtered through a 5-μm centrifugal filter. Bounded proteins were eluted through boiling with a 5X SDS loading dye.

### Labeling of active caspase-1

Around 2 × 10^6^ THP-1 cells/ml were seeded in a 24-well cell culture plate and differentiated for 3 days with PMA. The rB7 or rOff was added to the cells, and stimulation was performed as described above. Glycine was added to a cell culture medium simultaneously with LPS priming. Prior to stimulation with nigericin for 1 h, a 10 mM stock solution of biotin-VAD-fmk (Abcam) was added and incubated for 30 min, followed by further addition of a 1/10 medium volume of DPBS containing 10% IGEPAL, protease and phosphatase inhibitor cocktail, and Benzonase. The plate was incubated on ice for 5 min, and further incubated for 4 h at 4 °C after a streptavidin-conjugated dynabead was added. Bound active caspase-1 was washed three times with cold DPBS containing 1% IGEPAL and resuspended in the same buffer. The active caspase-1 was eluted by boiling with a 5X SDS loading dye and subjected to a western blot analysis.

### Reporting summary

Further information on research design is available in the Nature Research Reporting Summary linked to this article.

## Supplementary information

Supplemental material

Reporting Summary

## Data Availability

The crystal structure of ASC^PYD^ in complex with rB7 was deposited in the Protein Data Bank under accession code PDB 7E5B. Other data supporting the findings of this study are available within the paper and its supporting information file or from the corresponding authors upon reasonable request.
