# Correction: Oligomeric states of ASC specks regulate inflammatory responses by inflammasome in the extracellular space

**DOI:** 10.1038/s41420-023-01468-0

**Published:** 2023-07-04

**Authors:** Tae-Geun Yu, Jeong Seok Cha, Gijeong Kim, Yoo-Kyoung Sohn, Youngki Yoo, Uijin Kim, Ji-Joon Song, Hyun-Soo Cho, Hak-Sung Kim

**Affiliations:** 1 Departement of Biological Sciences, Korea Advanced Institute of Science and Technology (KAIST), Daejeon, 34141 Korea; 2 Department of Systems Biology, College of Life Science and Biotechnology, Yonsei University, Seoul, 03722 Korea; 3 Present Address: Research Institute of Pharmacy, Chung-Ang University, Seoul, 06974 Korea; 4 Present Address: R&D Center, Sugentech, Inc., Daejeon, Korea

**Keywords:** Structural biology, Inflammasome, Proteases, Prions, Protein design

Correction to: *Cell Death Discovery* 10.1038/s41420-023-01438-6, published online 29 April 2023

The original version of this article contained a mistake. Due to an error in production Hyun-Soo Cho was omitted to be named as a corresponding author. We apologize for the mistake. The original article has been corrected.

