# Supplementary material for: Oligomeric states of ASC specks regulate inflammatory responses by inflammasome in the extracellular space
Source: Cell Death Discov. 2023 Apr 29;9:142. doi: 10.1038/s41420-023-01438-6 (PMC10148886; doi:10.1038/s41420-023-01438-6)

Supplemental Material for

Oligomeric states of ASC specks regulate inflammatory responses by inflammasome in the extracellular space

Tae-Geun Yu, Jeong Seok Cha, Gijeong Kim, Yoo-Kyoung Sohn, Youngki Yoo, Uijin Kim, Ji-Joon Song, Hyun-Soo Cho, and Hak-Sung Kim

*Corresponding author. Email: hskim76@kaist.ac.kr

**This file includes:**

Figs. S1 to S12

Tables. S1 to S2


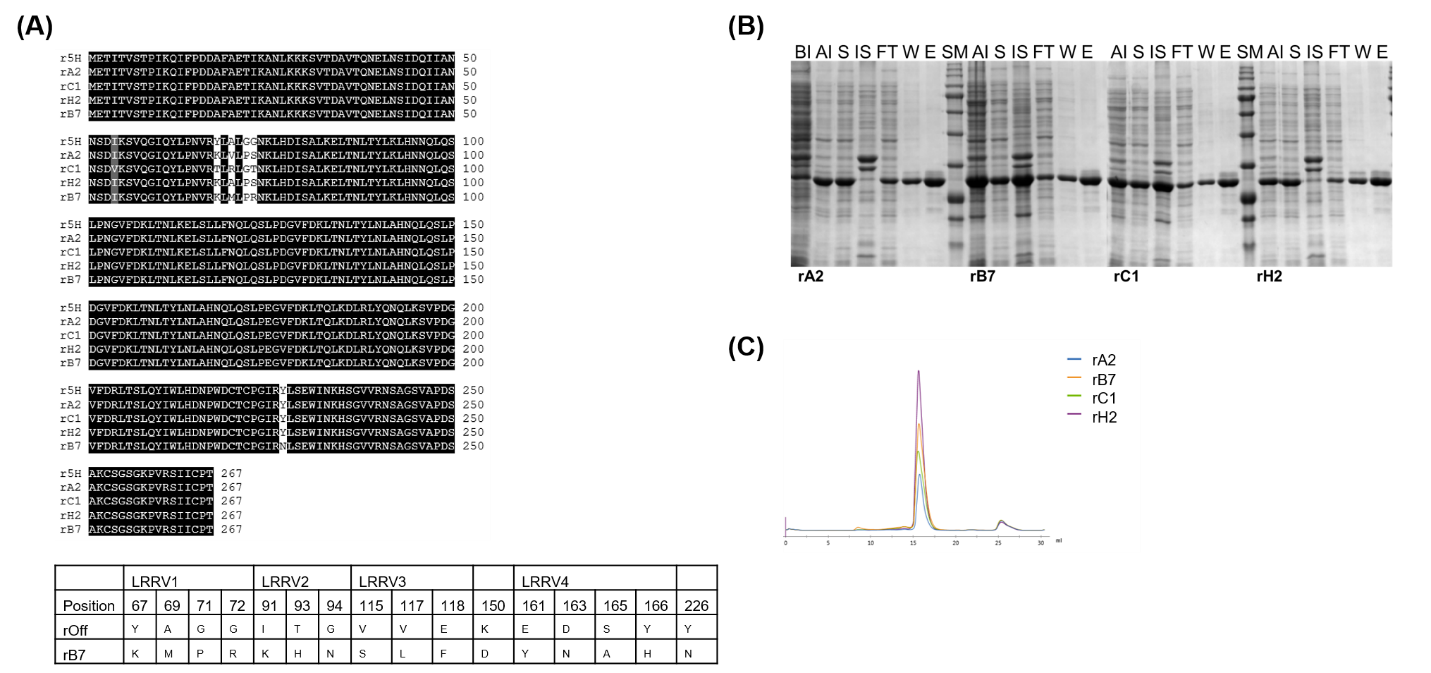


Fig. S1. Selection and affinity maturation of an ASC^PYD^-specific protein binder.

(A) Amino acids sequence alignments of an initially selected binder (r5H) and affinity-matured ones (rA2, rC1, rH2 and rB7). Amino acid residues at specific sites on the modules are shown for an off-target protein binder (rOff) and final protein binder (rB7). (B) SDS/PAGE analysis of protein binders during purification step. BI: before induction, AI: after induction, S: soluble, IS: insoluble, FT: flow through, W: washing, E: elution, SM: size marker. (C) Elution profiles of purified protein binders by FPLC (Superdex 200 Increase 10/300 GL, Cytiva).

Fig. S2. Uncropped western-blot images of Fig. 2.


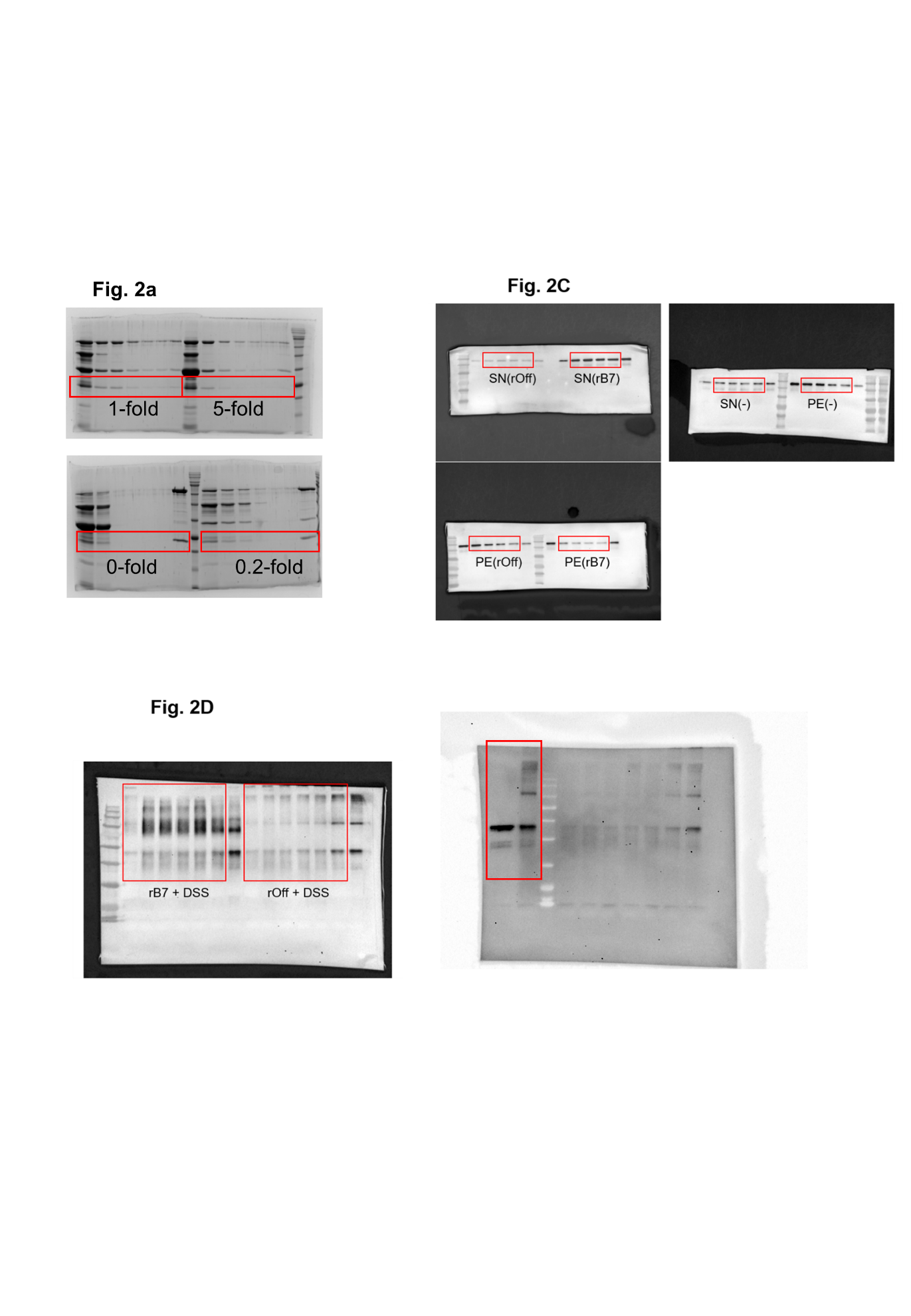


**
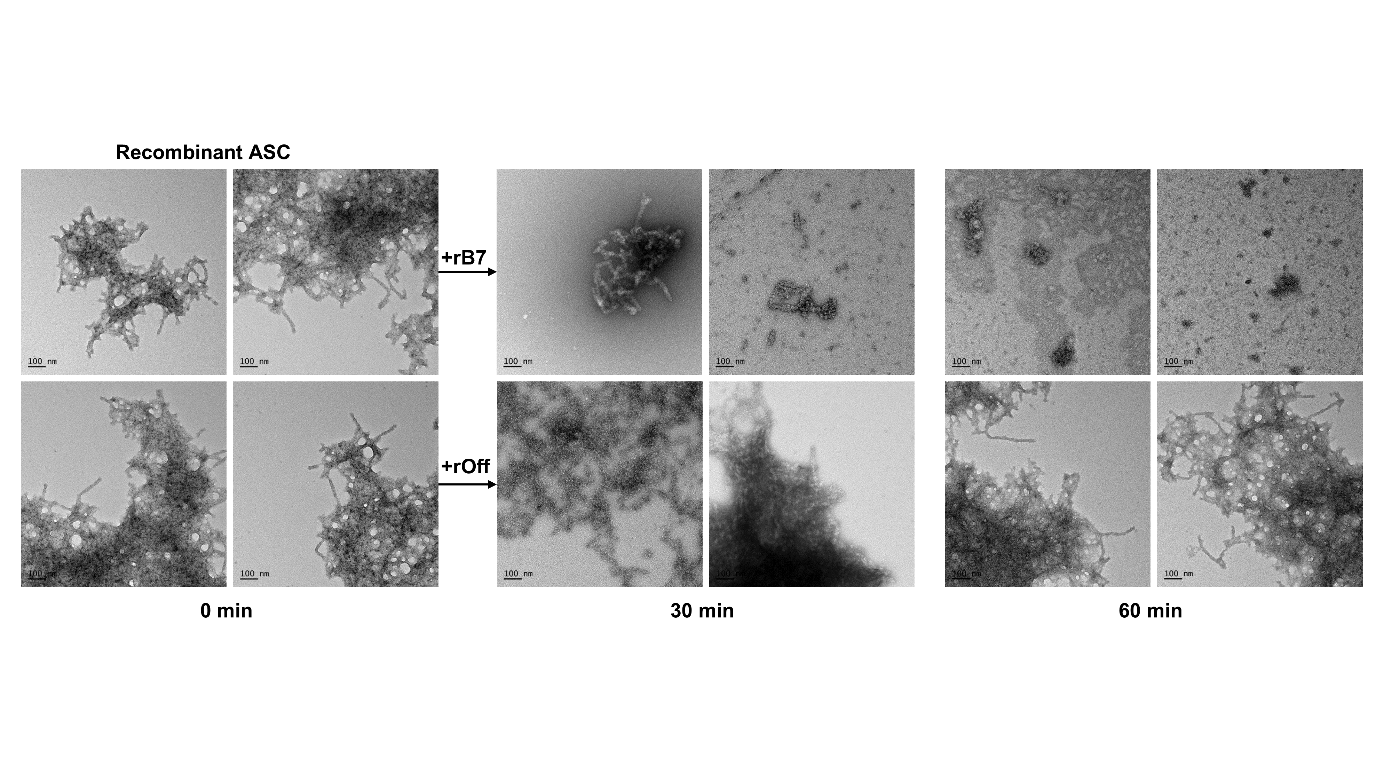
**

Fig. S3. Disassembly of ASC specks by rB7.

TEM images of recombinant ASC specks treated with rB7 or rOff at the concentration of 15 μg/ml over time.

**
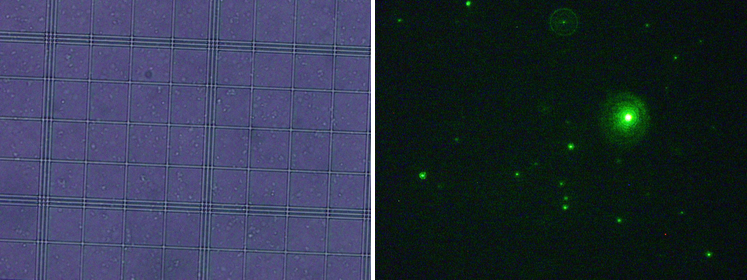
**

Fig. S4. Fluorescence imaging of mNeonGreen-fused full-length ASC specks.

mNeonGreen-fused ASC specks were expressed in HEK293 cells, followed by purification, and loaded on hemocytometer for imaging (left: bright field, right: fluorescence).


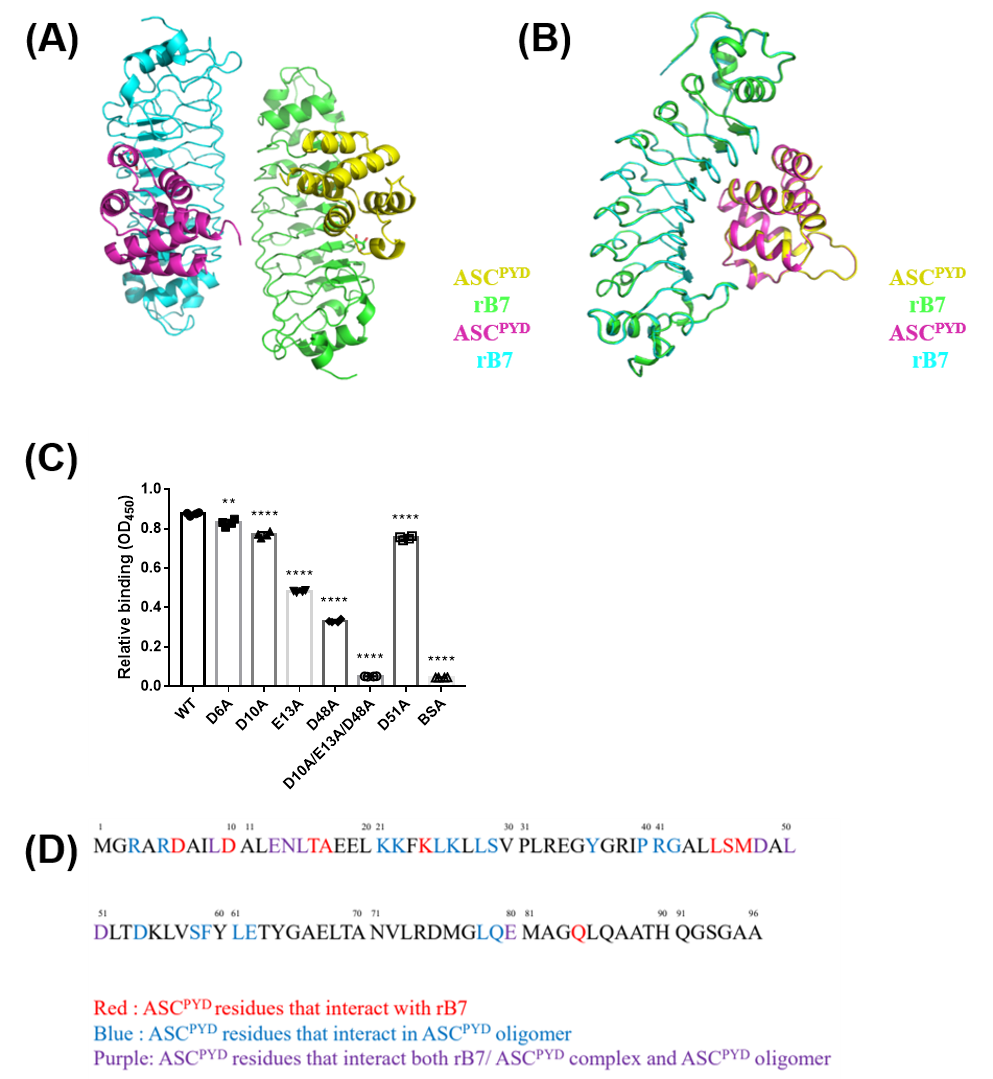


Fig. S5. Structural analysis of rB7 in complex with ASC^PYD^.

(A) The overall structure of rB7 in complex with ASC^PYD^. rB7 and ASC^PYD^ are presented in cartoon model. Two protomers of ASC^PYD^ and rB7 complex are in the asymmetric unit. (B) Superimpose of two protomers of rB7 in complex with ASC^PYD^. rB7 and ASC^PYD^ are presented in cartoon model. Two protomers are structurally identical based on RMSD of 0.237 Å. (C) Relative binding affinities of rB7 and MBP-fused ASC^PYD^ wild type and various mutants by ELISA. The data represent the means ± SDs from quadruplicate experiments. ^**^*p* < 0.01, ^****^*p* < 0.0001 compared with the control (two-tailed unpaired Student’s *t* test). (D) Amino acid residues on ASC^PYD^ which interact with rB7 and ASC^PYD^ oligomer. All ASC^PYD^ residues are represented, and a color description of each residue is described.


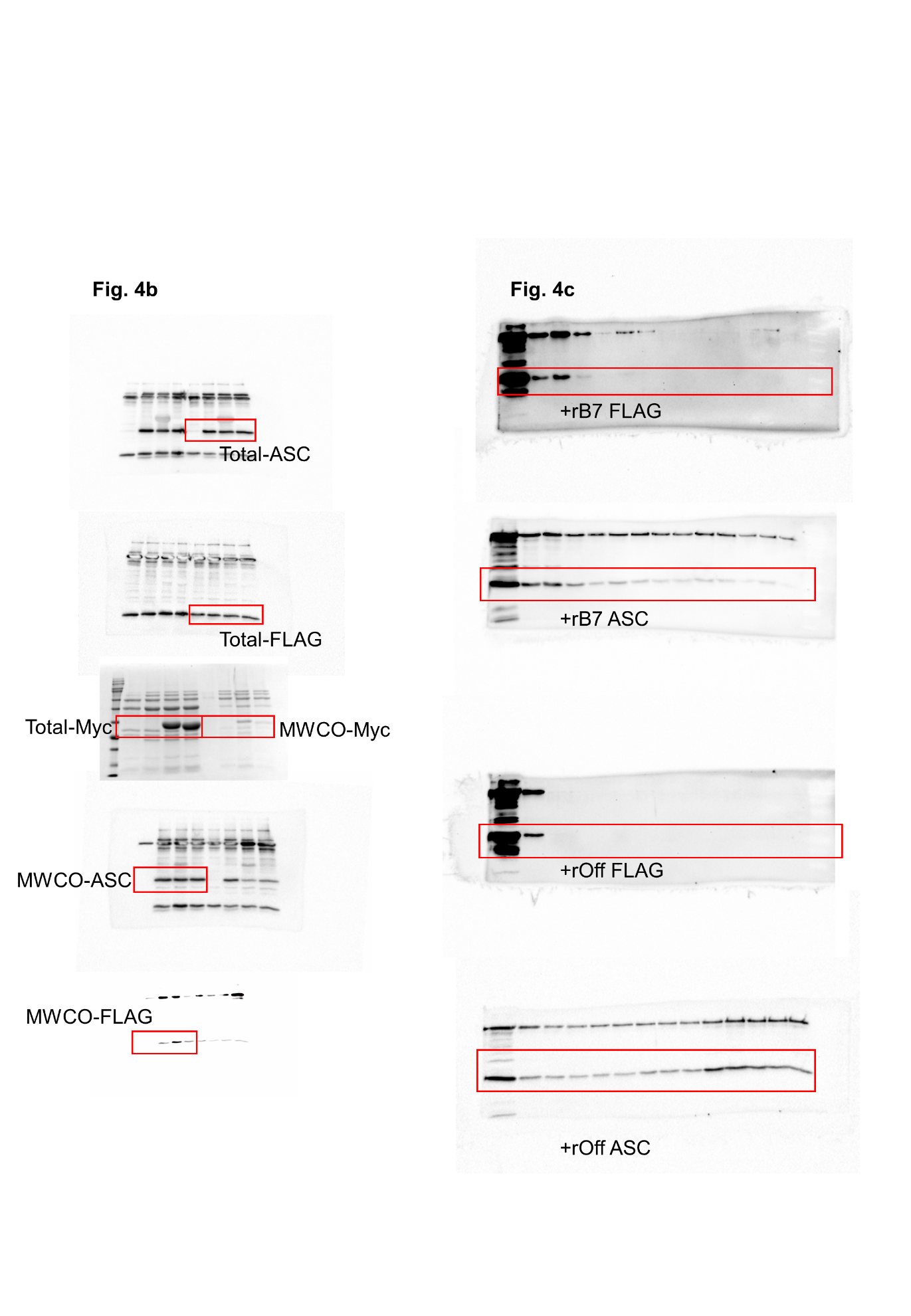


Fig. S6. Uncropped western-blot images of Fig. 4.


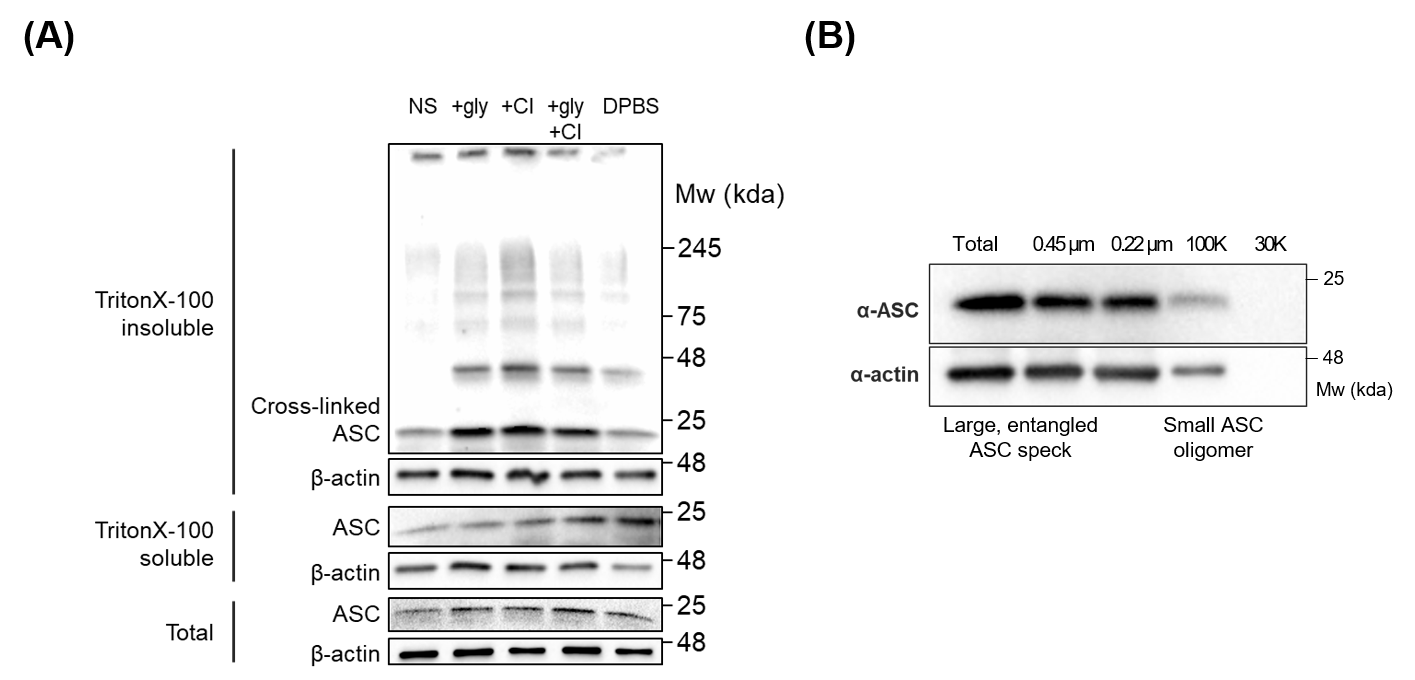


Fig. S7. Disassembly of ASC specks in extracellular space after cell lysis.

(A) Western blot analysis of cross-linked ASCs in the presence and absence of cell lysis during pyroptosis. PMA-differentiated THP-1 cells were stimulated with LPS/nigericin in the presence of 5 mM glycine (gly) and 50 μM caspase inhibitor (CI) to prevent cell lysis. 2 mM DSS was added 30 min after stimulation with nigericin to capture the oligomeric states of ASC, and the cells were directly lysed with 1% Triton X-100 followed by centrifugation (8000 g, 20 min). Separated supernatant (Triton X-100 soluble) and pellet (Triton X-100 insoluble) were analyzed by western blot. Oligomeric states of ASC decreased when cell lysis was not inhibited. The band with high molecular weight in NS may come from non-specific cross-linking. (B) Analysis of ASC specks with various sizes in extracellular space. Cell-free supernatants from stimulated THP-1 cells were filtered through different methods (0.45 μm syringe filter, 0.22 μm syringe filter, 100K amicon centrifugal filter, and 30K amicon centrifugal filter (Millipore)), followed by western blot analysis.


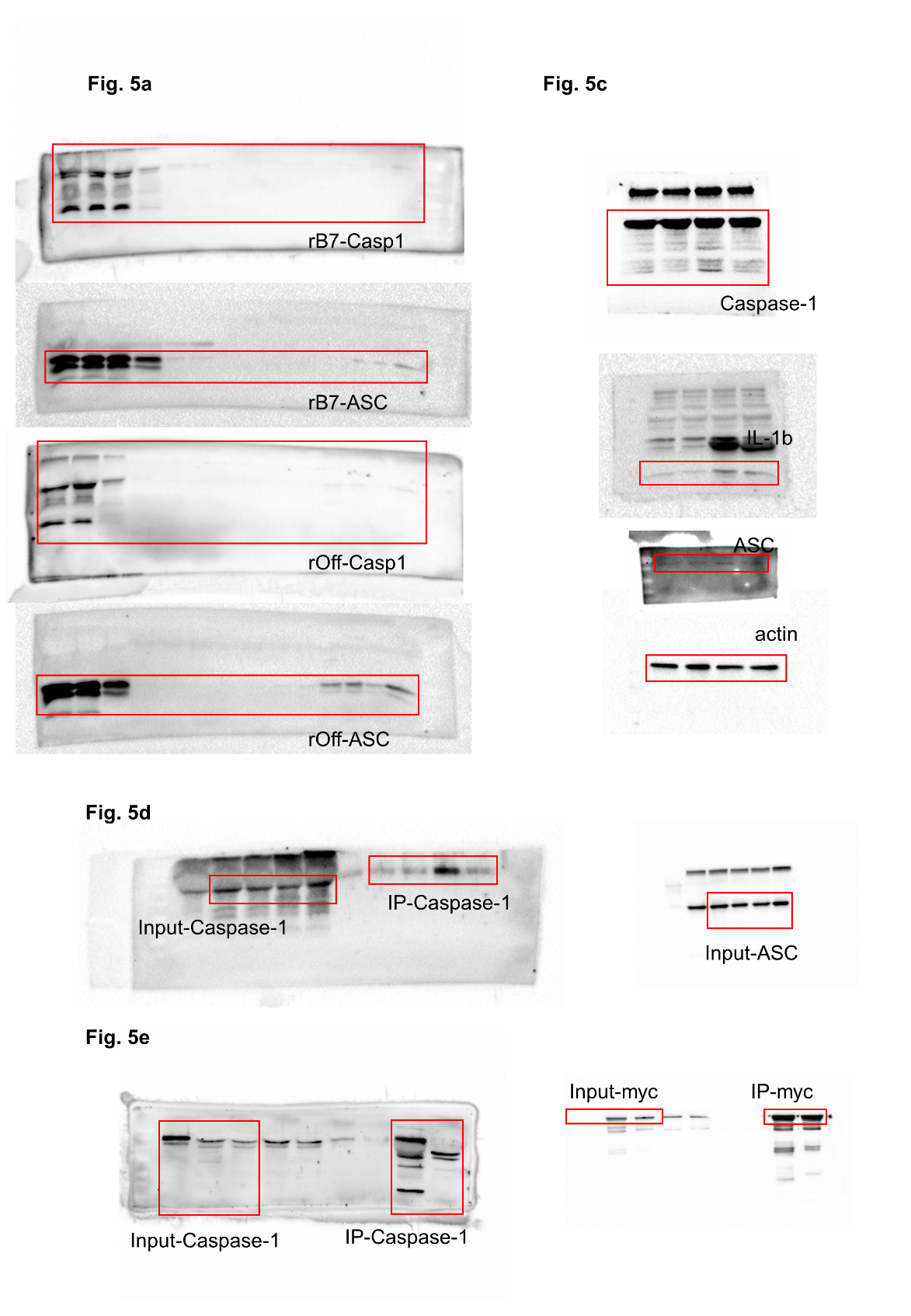


Fig. S8. Uncropped western-blot images of Fig. 5.


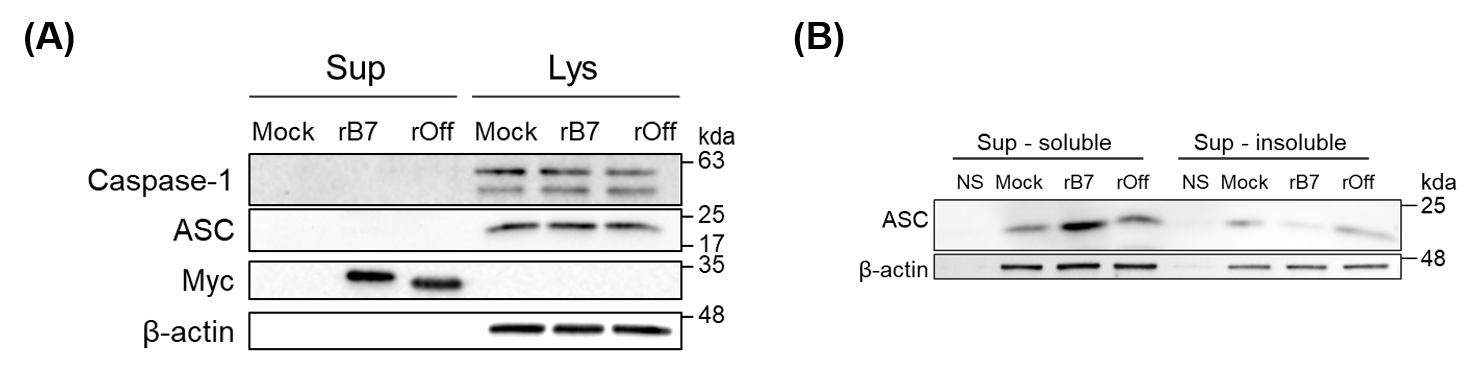


Fig. S9. Action of extracellularly treated rB7.

(A) Distribution of extracellularly treated rB7 or rOff between the supernatants and cell lysates. PMA-differentiated THP-1 cells were treated with rB7 or rOff, and distributions of rB7 and rOff between the supernatants and cell lysates were analyzed after 4 hrs of incubation. Anti-Myc tag antibody was used for the detection of a Myc tag-fused rB7 and rOff. ‘Sup’ and ‘Lys’ indicate the supernatants and cell lysates, respectively. (B) Disassembly of extracellular ASC specks by the action of rB7. THP-1 cells were stimulated with LPS/nigericin in the presence of rB7 or rOff, and the cell-free supernatants were separated into soluble and insoluble fractions by centrifugation (18000 g, 10 min). The ASC levels in soluble and insoluble fractions were analyzed by western blot. NS indicates non-stimulated cells.


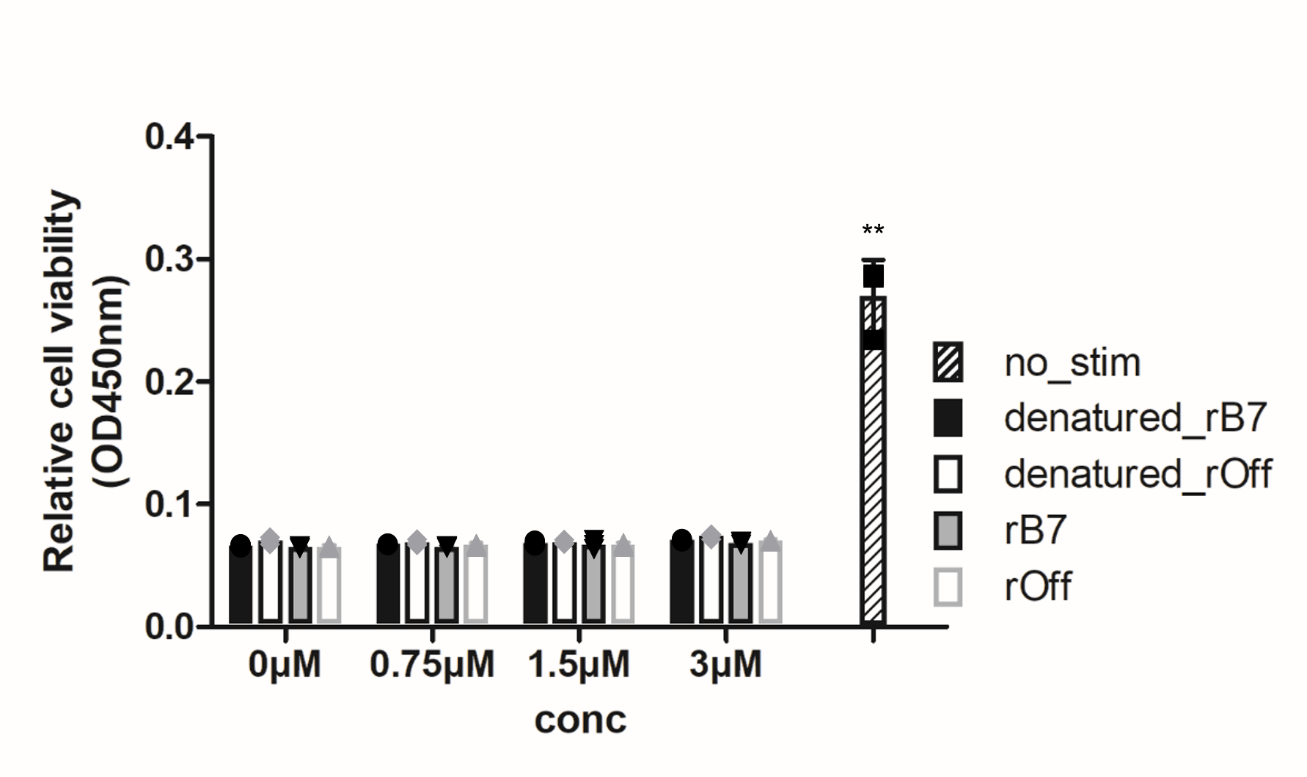


Fig. S10. The viability of stimulated THP-1 cells in the presence of rB7.

THP-1 cells were treated with rB7 or rOff, followed by stimulation with LPS/nigericin, and the cell viability was measured by cck-8 assay. Both rB7 and rOff had a negligible effect on pyroptotic cell death. ^**^*p* < 0.01 compared with the control (two-tailed unpaired Student’s *t* test).


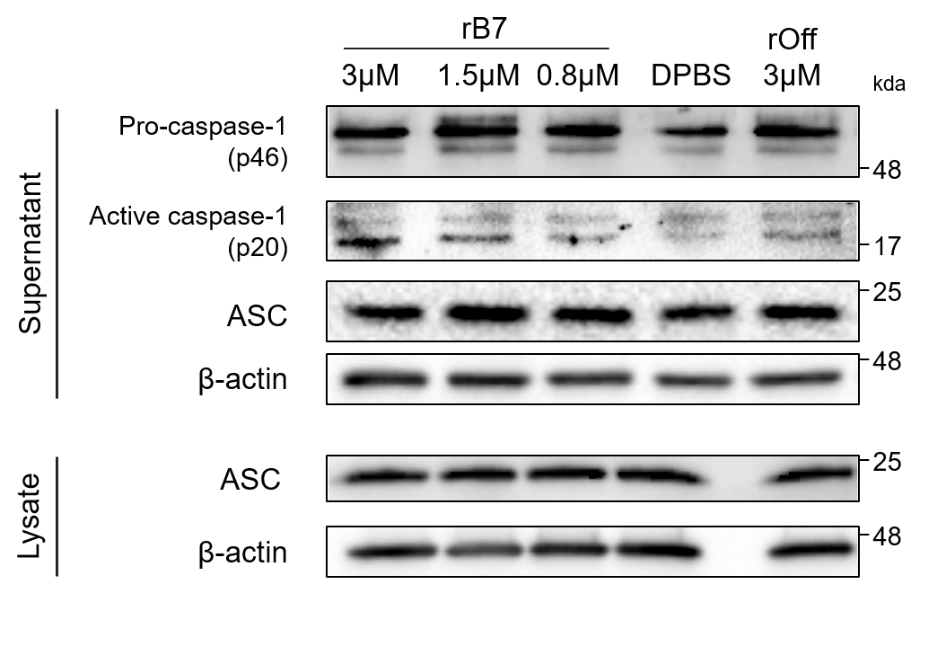


Fig. S11. Western blot analysis of stimulated THP-1 in the presence of rB7.

THP-1 cells were stimulated with LPS/nigericin in the presence of rB7 or rOff. Cell-free supernatants were obtained by centrifugation (600 g, 5 min), and separated cells were lysed by RIPA buffer (Thermo Scientific). The levels of ASC, β-actin and caspase species (p46 and p20) were analyzed by western blot. Concentrations of rB7 and rOff are indicated.


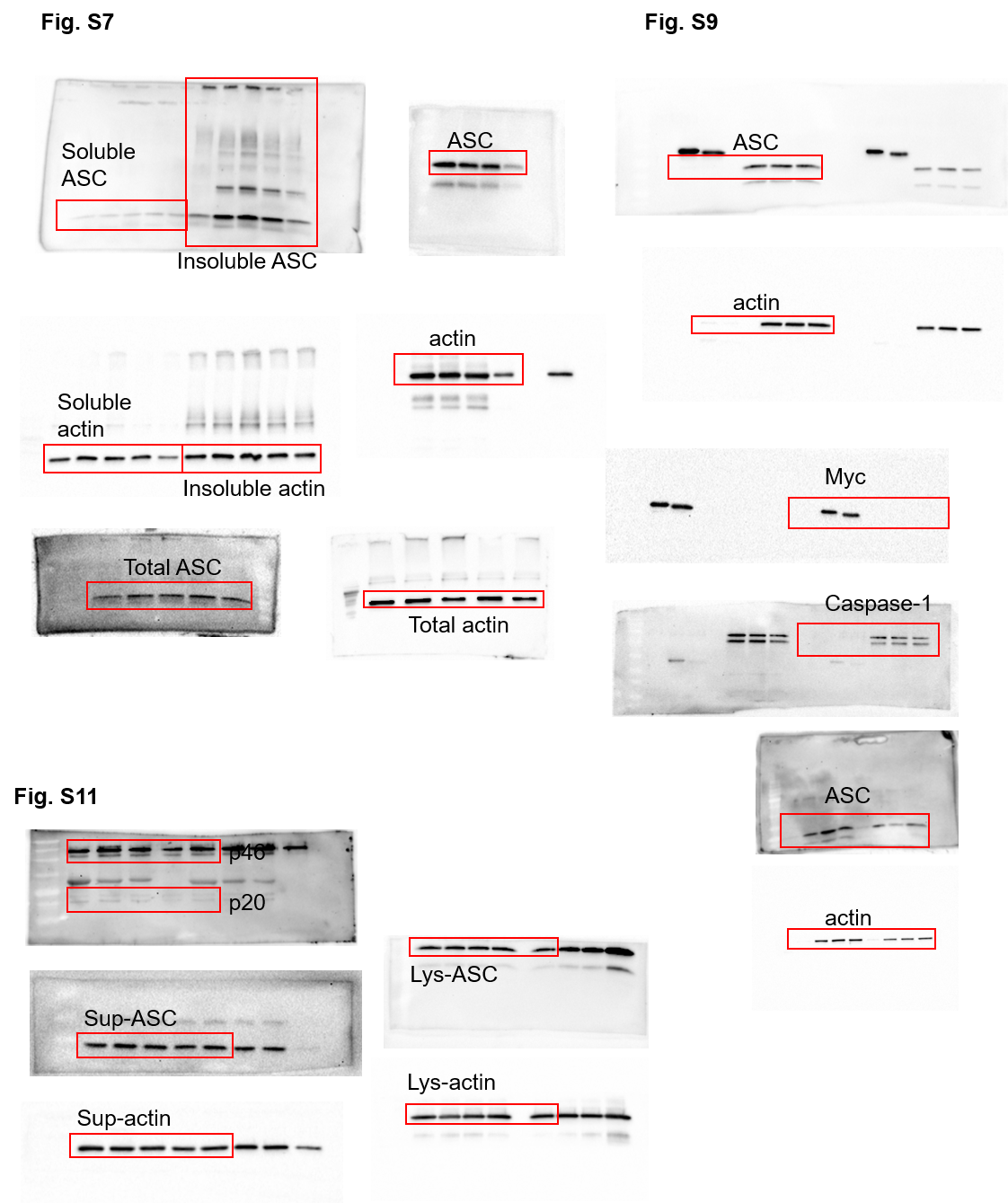


Fig. S12. Uncropped western-blot images of Fig. S7, Fig. S9, and Fig. S11.

Table. S1. Protein sequences of constructs used in this study.

| **Clone** | **Sequence** |
| --- | --- |
| **MBP-ASC^PYD^**  **(1-106)** | MGTSHHHHHHHHHHKIEEGKLVIWINGDKGYNGLAEVGKKFEKDTGIKVTVEHPDKLEEKFPQVAATGDGPDIIFWAHDRFGGYAQSGLLAEITPDKAFQDKLYPFTWDAVRYNGKLIAYPIAVEALSLIYNKDLLPNPPKTWEEIPALDKELKAKGKSALMFNLQEPYFTWPLIAADGGYAFKYENGKYDIKDVGVDNAGAKAGLTFLVDLIKNKHMNADTDYSIAEAAFNKGETAMTINGPWAWSNIDTSKVNYGVTVLPTFKGQPSKPFVGVLSAGINAASPNKELAKEFLENYLLTDEGLEAVNKDKPLGAVALKSYEEELAKDPRIAATMENAQKGEIMPNIPQMSAFWYAVRTAVINAASGRQTVDEALKDAQTGTDYDIPTTENLYFQGHMGRARDAILDALENLTAEELKKFKLKLLSVPLREGYGRIPRGALLSMDALDLTDKLVSFYLETYGAELTANVLRDMGLQEMAGQLQAATHQGSGAAPAGIQAPPQSHMIY |
| **MBP-ASC^PYD^**  **(1-96)** | MGTSHHHHHHHHHHKIEEGKLVIWINGDKGYNGLAEVGKKFEKDTGIKVTVEHPDKLEEKFPQVAATGDGPDIIFWAHDRFGGYAQSGLLAEITPDKAFQDKLYPFTWDAVRYNGKLIAYPIAVEALSLIYNKDLLPNPPKTWEEIPALDKELKAKGKSALMFNLQEPYFTWPLIAADGGYAFKYENGKYDIKDVGVDNAGAKAGLTFLVDLIKNKHMNADTDYSIAEAAFNKGETAMTINGPWAWSNIDTSKVNYGVTVLPTFKGQPSKPFVGVLSAGINAASPNKELAKEFLENYLLTDEGLEAVNKDKPLGAVALKSYEEELAKDPRIAATMENAQKGEIMPNIPQMSAFWYAVRTAVINAASGRQTVDEALKDAQTGTDYDIPTTENLYFQGHMGRARDAILDALENLTAEELKKFKLKLLSVPLREGYGRIPRGALLSMDALDLTDKLVSFYLETYGAELTANVLRDMGLQEMAGQLQAATHQGSGAA |
| **MBP-ASC full length** | MGTSHHHHHHHHHHKIEEGKLVIWINGDKGYNGLAEVGKKFEKDTGIKVTVEHPDKLEEKFPQVAATGDGPDIIFWAHDRFGGYAQSGLLAEITPDKAFQDKLYPFTWDAVRYNGKLIAYPIAVEALSLIYNKDLLPNPPKTWEEIPALDKELKAKGKSALMFNLQEPYFTWPLIAADGGYAFKYENGKYDIKDVGVDNAGAKAGLTFLVDLIKNKHMNADTDYSIAEAAFNKGETAMTINGPWAWSNIDTSKVNYGVTVLPTFKGQPSKPFVGVLSAGINAASPNKELAKEFLENYLLTDEGLEAVNKDKPLGAVALKSYEEELAKDPRIAATMENAQKGEIMPNIPQMSAFWYAVRTAVINAASGRQTVDEALKDAQTGTDYDIPTTENLYFQGHMGRARDAILDALENLTAEELKKFKLKLLSVPLREGYGRIPRGALLSMDALDLTDKLVSFYLETYGAELTANVLRDMGLQEMAGQLQAATHQGSGAAPAGIQAPPQSAAKPGLHFIDQHRAALIARVTNVEWLLDALYGKVLTDEQYQAVRAEPTNPSKMRKLFSFTPAWNWTCKDLLLQALRESQSYLVEDLERS |
| **ASC-mNG2** | MEQKLISEEDLSRGSEFDPGAMGRARDAILDALENLTAEELKKFKLKLLSVPLREGYGRIPRGALLSMDALDLTDKLVSFYLETYGAELTANVLRDMGLQEMAGQLQAATHQGSGAAPAGIQAPPQSAAKPGLHFIDQHRAALIARVTNVEWLLDALYGKVLTDEQYQAVRAEPTNPSKMRKLFSFTPAWNWTCKDLLLQALRESQSYLVEDLERSCGLDGTAGPGSIATMVSKGEEDNMASLPATHELHIFGSINGVDFDMVGQGTGNPNDGYEELNLKSTKGDLQFSPWILVPHIGYGFHQYLPYPDGMSPFQAAMVDGSGYQVHRTMQFEDGASLTVNYRYTYEGSHIKGEAQVMGTGFPADGPVMTNTLTAADWCMSKKTYPNDKTIISTFKWSYTTVNGKRYRSTARTTYTFAKPMAANYLKNQPMYVFRKTELKHSKTEVNFKEWVKSFTD |
| **rOff**  **(WT)** | METITVSTPIKQIFPDDAFAETIKANLKKKSVTDAVTQNELNSIDQIIANNSDIKSVQGIQYLPNVRYLALGGNKLHDISALKELTNLTYLILTGNQLQSLPNGVFDKLTNLKELVLVENQLQSLPDGVFDKLTNLTYLNLAHNQLQSLPKGVFDKLTNLTELDLSYNQLQSLPEGVFDKLTQLKDLRLYQNQLKSVPDGVFDRLTSLQYIWLHDNPWDCTCPGIRYLSEWINKHSGVVRNSAGSVAPDSAKCSGSGKPVRSIICPT |
| **7G**  **(1^st^ library)** | METITVSTPIKQIFPDDAFAETIKANLKKKSVTDAVTQNELNSIDQIIANNSDIKSVQGIQYLPNVRYLALGGNKLHDISALKELTNLTYLNLKRNQLQSLPNGVFDKLTNLKELRLFHNQLQSLPDGVFDKLTNLTYLNLAHNQLQSLPKGVFDKLTNLTELDLSYNQLQSLPDGVFDRLTSLQYIWLHDNPWDCTCPGIRYLSEWINKHSGVVRNSAGSVAPDSAKCSGSGKPVRSIICPT |
| **5H**  **(1^st^ library)** | METITVSTPIKQIFPDDAFAETIKANLKKKSVTDAVTQNELNSIDQIIANNSDIKSVQGIQYLPNVRYLALGGNKLHDISALKELTNLTYLKLHNNQLQSLPNGVFDKLTNLKELSLLFNQLQSLPDGVFDKLTNLTYLNLAHNQLQSLPDGVFDKLTNLTYLNLAHNQLQSLPEGVFDKLTQLKDLRLYQNQLKSVPDGVFDRLTSLQYIWLHDNPWDCTCPGIRYLSEWINKHSGVVRNSAGSVAPDSAKCSGSGKPVRSIICPT |
| **A2**  **(2^nd^ library)** | METITVSTPIKQIFPDDAFAETIKANLKKKSVTDAVTQNELNSIDQIIANNSDIKSVQGIQYLPNVRKLVLPSNKLHDISALKELTNLTYLKLHNNQLQSLPNGVFDKLTNLKELSLLFNQLQSLPDGVFDKLTNLTYLNLAHNQLQSLPDGVFDKLTNLTYLNLAHNQLQSLPEGVFDKLTQLKDLRLYQNQLKSVPDGVFDRLTSLQYIWLHDNPWDCTCPGIRYLSEWINKHSGVVRNSAGSVAPDSAKCSGSGKPVRSIICPT |
| **H2**  **(2^nd^ library)** | METITVSTPIKQIFPDDAFAETIKANLKKKSVTDAVTQNELNSIDQIIANNSDIKSVQGIQYLPNVRKLALPSNKLHDISALKELTNLTYLKLHNNQLQSLPNGVFDKLTNLKELSLLFNQLQSLPDGVFDKLTNLTYLNLAHNQLQSLPDGVFDKLTNLTYLNLAHNQLQSLPEGVFDKLTQLKDLRLYQNQLKSVPDGVFDRLTSLQYIWLHDNPWDCTCPGIRYLSEWINKHSGVVRNSAGSVAPDSAKCSGSGKPVRSIICPT |
| **C1**  **(2^nd^ library)** | METITVSTPIKQIFPDDAFAETIKANLKKKSVTDAVTQNELNSIDQIIANNSDVKSVQGIQYLPNVRTLRLGTNKLHDISALKELTNLTYLKLHNNQLQSLPNGVFDKLTNLKELSLLFNQLQSLPDGVFDKLTNLTYLNLAHNQLQSLPDGVFDKLTNLTYLNLAHNQLQSLPEGVFDKLTQLKDLRLYQNQLKSVPDGVFDRLTSLQYIWLHDNPWDCTCPGIRYLSEWINKHSGVVRNSAGSVAPDSAKCSGSGKPVRSIICPT |
| **B7**  **(2^nd^ library)** | METITVSTPIKQIFPDDAFAETIKANLKKKSVTDAVTQNELNSIDQIIANNSDIKSVQGIQYLPNVRKLMLPRNKLHDISALKELTNLTYLKLHNNQLQSLPNGVFDKLTNLKELSLLFNQLQSLPDGVFDKLTNLTYLNLAHNQLQSLPDGVFDKLTNLTYLNLAHNQLQSLPEGVFDKLTQLKDLRLYQNQLKSVPDGVFDRLTSLQYIWLHDNPWDCTCPGIRNLSEWINKHSGVVRNSAGSVAPDSAKCSGSGKPVRSIICPT |
| **rB7-myc-his** | METITVSTPIKQIFPDDAFAETIKANLKKKSVTDAVTQNELNSIDQIIANNSDIKSVQGIQYLPNVRKLMLPRNKLHDISALKELTNLTYLKLHNNQLQSLPNGVFDKLTNLKELSLLFNQLQSLPDGVFDKLTNLTYLNLAHNQLQSLPDGVFDKLTNLTYLNLAHNQLQSLPEGVFDKLTQLKDLRLYQNQLKSVPDGVFDRLTSLQYIWLHDNPWDCTCPGIRNLSEWINKHSGVVRNSAGSVAPDSAKCSGSGKPVRSIICPTEQKLISEEDLLEHHHHHH |
| **rOff-myc-his** | METITVSTPIKQIFPDDAFAETIKANLKKKSVTDAVTQNELNSIDQIIANNSDIKSVQGIQYLPNVRYLALGGNKLHDISALKELTNLTYLILTGNQLQSLPNGVFDKLTNLKELVLVENQLQSLPDGVFDKLTNLTYLNLAHNQLQSLPKGVFDKLTNLTELDLSYNQLQSLPEGVFDKLTQLKDLRLYQNQLKSVPDGVFDRLTSLQYIWLHDNPWDCTCPGIRYLSEWINKHSGVVRNSAGSVAPDSAKCSGSGKPVRSIICPTEQKLISEEDLLEHHHHHH |

Table. S2. Parameters for crystal structure of ASC^PYD^ and rB7.


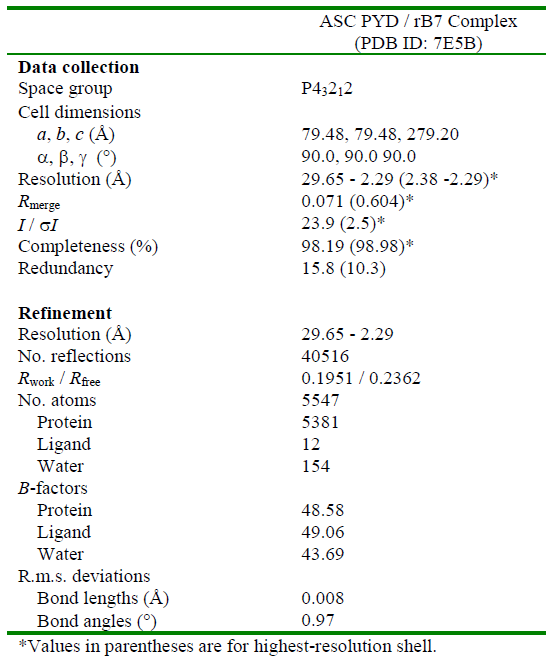

Supplement: Supplementary file 1 — Supplemental material [file 41420_2023_1438_MOESM1_ESM.docx]
